# Dereplication Guided Discovery of Secondary Metabolites of Mixed Biosynthetic Origin from *Aspergillus aculeatus*

**DOI:** 10.3390/molecules190810898

**Published:** 2014-07-25

**Authors:** Lene M. Petersen, Casper Hoeck, Jens C. Frisvad, Charlotte H. Gotfredsen, Thomas O. Larsen

**Affiliations:** 1 Chemodiversity Group, Department of Systems Biology, Søltofts Plads B221, Technical University of Denmark, Kgs. Lyngby DK-2800, Denmark; E-Mails: lmape@bio.dtu.dk (L.M.P.); jcf@bio.dtu.dk (J.C.F.); 2 Department of Chemistry, Kemitorvet B201, Technical University of Denmark, Kgs. Lyngby DK-2800, Denmark; E-Mails: casho@kemi.dtu.dk (C.H.); chg@kemi.dtu.dk (C.H.G.)

**Keywords:** *Aspergillus aculeatus*, Aspergilli, natural products, secondary metabolism, dereplication, sclerotia

## Abstract

Investigation of the chemical profile of the industrially important black filamentous fungus *Aspergillus aculeatus* by UHPLC-DAD-HRMS and subsequent dereplication has led to the discovery of several novel compounds. Isolation and extensive 1D and 2D NMR spectroscopic analyses allowed for structural elucidation of a dioxomorpholine, a unique okaramine, an aflavinine and three novel structures of mixed biosynthetic origin, which we have named aculenes A–C. Moreover, known analogues of calbistrins, okaramines and secalonic acids were detected. All novel compounds were tested for antifungal activity against *Candida albicans*, however all showed only weak or no activity. *Aspergillus aculeatus* IBT 21030 was additionally shown to be capable of producing sclerotia. Examination of the sclerotia revealed a highly regulated production of metabolites in these morphological structures.

## 1. Introduction

*Aspergillus aculeatus* is a filamentous fungus belonging to *Aspergillus* section *Nigri*—the black aspergilli. At least 145 metabolites have been characterized from the black aspergilli [1], many of which are biologically active. Naphtho-γ-pyrones (NGPs) such as aurasperone A and rubrofusarin B from *A. niger* are known to be antifungal [2], while other NGPs are reported to have antitumor activities [3]. Mycotoxins produced by *A. niger*, such as ochratoxin A [4] or the fumonisins [5] are also known.

The black aspergilli can be divided into different clades. *A. aculeatus* belongs to the uniseriate black aspergilli and is closely related to *A. aculeatinus*, *A. uvarum*, *A. japonicus*, *A. fijiensis*, *A. trinidadensis*, *A. floridensis*, *A. brunneoviolaceus* and *A. violaceofuscus* [6,7]. These fungi differ from the other black aspergilli in their morphology, physiological behavior and in the production of secondary metabolites. The fungi belonging to this group can produce secondary metabolites, which can be both polyketide (PK) and nonribosomal peptide (NRP) derived or of mixed biosynthetic origin [1]. Several metabolites, including aculeacins A–G [8,9], CJ-15,183 [10], secalonic acids D and F [11] and okaramines H and I [12], have been reported in fungi identified as *A. aculeatus*. Furthermore, asperaculin A [13], aspergillusol A [14] and aculeatusquinones A–D [15] have been reported in marine strains of *A. aculeatus*. Different biological activities are reported for these metabolites, including antifungal (aculeacins A-G and CJ-15,183), enzyme inhibitory (CJ-15,183 and aspergilluson A), antimicrobial activities (secalonic D and F) and cytotoxicity (aculeatusquinone B and D).

While some black aspergilli are important in the biotechnological industry for production of enzymes and organic acids [16,17], some species can also be food and feed contaminants [18]. In fact, the black aspergilli are among the most common fungi connected to postharvest decay of fruit, beans and nuts, and *A. aculeatus* is no exception [18,19].

*A. aculeatus* is used to produce important industrial enzymes such as cellulases [20], xylanases [21,22] and proteases [23], which are used commercially in the food and feed industries. Moreover a marine strain of *A. aculeatus* has tested active against *Staphylococcus aureus* [24]. Based on both the industrial applications of *A. aculeatus* as well as food and feed contamination hazards it is of great importance to know what metabolites are being produced by this fungus. The aim of the current work has been to investigate the chemical profile of *A. aculeatus* seeking to discover novel compounds and to test for the antifungal activity of its metabolites.

## 2. Results and Discussion

**Figure 1:**
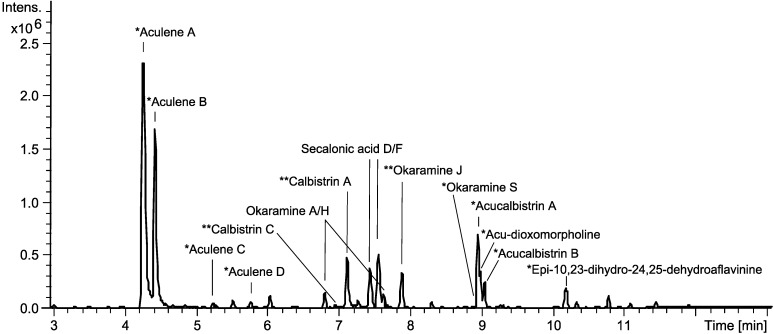
Base peak chromatogram illustrating the dereplication of some of the major compounds in the extract from *A. aculeatus* IBT 21030 as well as the results of purification and structural elucidation of novel compounds. Based on cultivation on YES media for 7 days at 25 °C in the dark. * Novel compounds reported here for the first time. ** Compounds reported from *A. aculeatus* for the first time.

Initial analysis of the chemistry of *A. aculeatus* involved two strains (IBT 21030 and IBT 3244) which were investigated on a series of solid media (YES, CYA, MEA, OAT and CREA) [25]. The strains were cultivated at 25 and 30 °C in the dark for 7 days and were investigated with micro-scale extractions [26]. The secondary metabolite profiles were analyzed with UHPLC-DAD-HRMS, which demonstrated that the strains showed highly similar chemical profiles. However, more metabolites were produced by the *A. aculeatus* IBT 21030 strain, hence this strain was chosen for further work. The medium used in the cultivations had varying effect on metabolite production. While cultivation on MEA, OAT and CREA did not lead to diverse production, cultivation on both CYA and YES lead to production of several different metabolites. The YES medium was selected for further work, and it was found that the optimal cultivation temperature was 25 °C. The *A. aculeatus* strain was inoculated on YES media and incubated at 25 °C and analyzed after 2, 4, 7, and 10 days to test the effect of incubation time on the metabolite production. These experiments showed that 7 days of incubation gave the optimum production of secondary metabolites. Based on these initial experiments, *A. aculeatus* was inoculated on 200 plates of YES and incubated at 25 °C for seven days in the dark. The results of the dereplication as well as isolation and elucidation of novel compounds are depicted in Figure 1.

**Figure 2:**
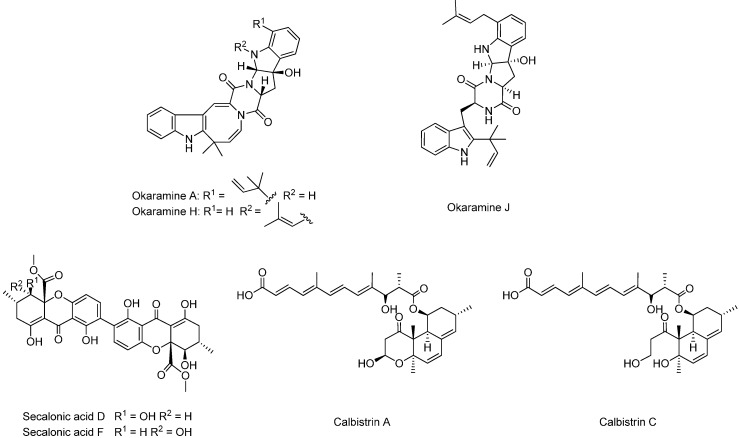
Known compounds dereplicated from *A. aculeatus*. Okaramines A and H as well as secalonic acids have previously been reported from the organism, whereas the calbistrins and okaramine J are reported from *A. aculeatus* for the first time.

Dereplication was performed by UV- and HRMS-data to identify known compounds. An in-house library of microbial metabolites [27] as well as the AntiBase 2012 natural products database [28] were used for identification. Many of the major peaks could be dereplicated as compounds already known to be produced by *A. aculeatus* (Figure 1 and Figure 2). This included secalonic acids D and F [11], as well as okaramines A and H [12]. One further okaramine was produced, which could not be unambiguously dereplicated, since the HRMS and UV data pointed towards either okaramine C, J *or* L. The compound was isolated and the structure was elucidated by 1D and 2D NMR and the structure was established to correspond to okaramine J, which has previously been described from *Penicillium ochrochloron* (formerly identified as *Penicillium simplicissimum*) [29,30].

Two calbistrin analogues could also be dereplicated from *A. aculeatus*. Calbistrins A–D, have been described from *Penicillium restrictum* by Jackson *et al.* [31] and the structures of the four compounds were later elucidated by the same group [32]. Calbistrin A is biologically active as an antifungal agent, a promoter of nerve growth factor production and a cholesterol lowering agent. Identification of the two calbistrins was not possible solely based on HRMS and DAD data, as calbistrins pairwise have the same molecular formula (A and B have the same molecular formula and likewise for calbistrins C and D). The two compounds were therefore isolated and their structures elucidated by 1D and 2D NMR, and it was shown that the two compounds produced by *A. aculeatus* were calbistrin A and C, Figure 2.

### 2.1. A Novel Class of Compounds, Named the aculenes, Has a Mixed Biosynthetic Origin

**Figure 3:**
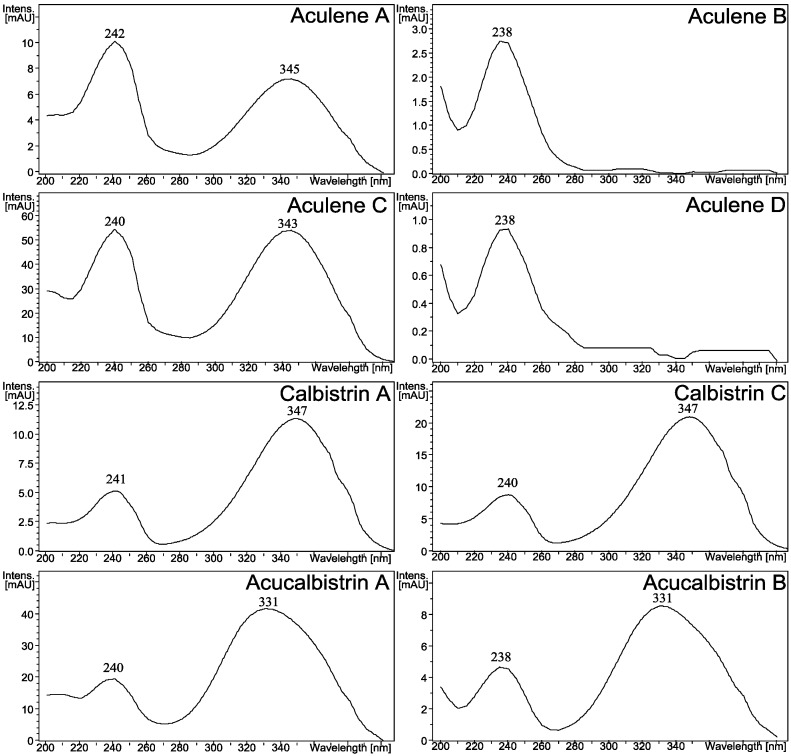
UV-spectra of selected *A. aculeatus* metabolites. The remaining UV spectra can be found in the Supporting Information.

The two major metabolites produced by *A. aculeatus*, aculenes A and B (Figure 1) have previously been detected by UHPLC-DAD-HRMS in the related black aspergillus *A. saccharolyticus* [33], but the structures have not been elucidated. The molecular formulae of the two compounds were determined by HRMS to be C_19_H_25_NO_3_ and C_19_H_27_NO_3_, respectively. The mass difference of 2 Da and the fact that the two compounds eluted very close to each other suggested that the compounds were related and only differed by a double bond. The UV-spectra differed greatly as depicted in Figure 3, indeed indicating aculene A to contain a more conjugated chromophore.

**Figure 4:**
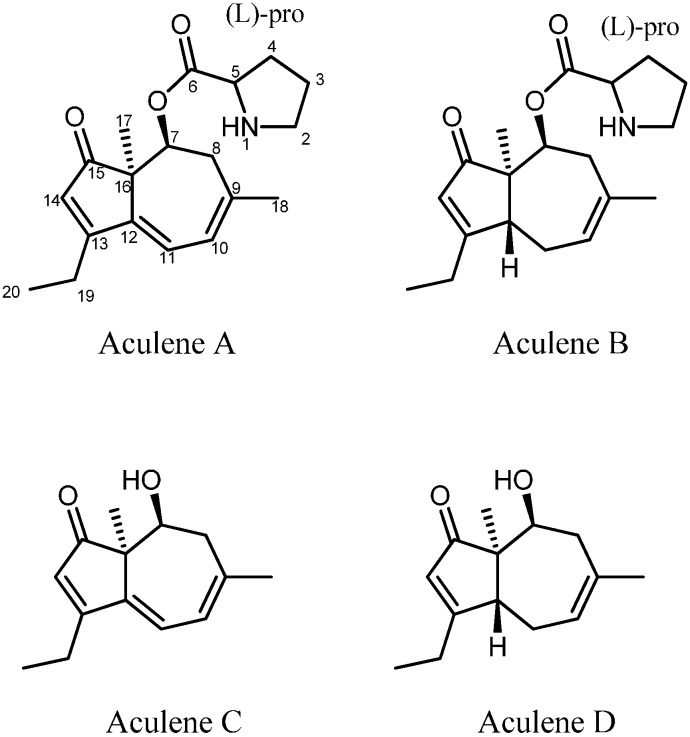
The structure of aculenes A–D. The structures of aculenes A–C have been verified by 1D and 2D NMR spectroscopy, whereas the structure of aculene D has been suggested based on UHPLC-DAD-HRMS data. The stereochemistry shown is relative.

**Figure 5:**
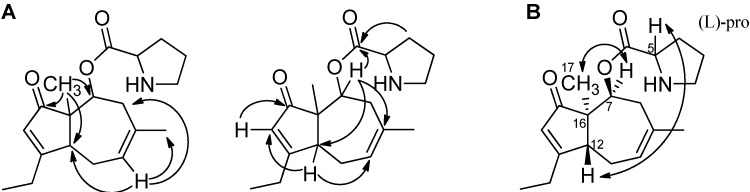
**A**. Important HMBC correlations in the elucidation of aculene B. **B**. The NOEs used to solve the stereochemistry. Similar HMBCs and NOEs were observed for aculenes A and C, see Table 1 and Supplementary Table S2.

The structures of the two compounds were elucidated by 1D- and 2D NMR spectroscopy and the structures of aculenes A and B are depicted in Figure 4. The conjugation in aculene A was in agreement with the recorded UV spectrum. The only difference with aculene B is the absent double bond between C-11 and C-12 and as seen, this structure was also in accordance with the obtained UV spectrum. The ^1^H-NMR spectra of both aculenes A and B revealed the presence of one H_α_ proton (H-5), which was found to be in a COSY spin system with three methylene groups (H-2, H-3 and H-4), which where elucidated as a proline ring. The remaining resonances in the ^1^H-NMR spectra belonged to a fused five and seven membered ring system. The linking between the COSY spin systems for this part and assignments of the quaternary carbons were accomplished through detailed analysis of the HMBC experimental data. Some of the important HMBC correlations for the elucidation of aculene B are depicted in Figure 5.

**Table 1:** ^1^H-NMR spectroscopic data (500 and 800 MHz, DMSO-*d_6_*) and HMBCs for aculenes A–C.

		Aculene A			Aculene B		Aculene C	
Position	δ_H_ (*J* in Hz)		HMBC		δ_H_ (*J* in Hz)	HMBC	δ_H_ (*J* in Hz)	HMBC
**1**		-		-	-	-		
**2**		3.15 (m)		-	3.21 (m)	3,4,5		
**2'**		3.09 (m)		-	3.14 (m)	3,4,5		
**3**		1.83 (m)		-	1.84 (m)	2,4,5		
**3'**		1.67 (m)		-	1.76 (m)	2,4,5		
**4**		1.94 (m)		2,6	2.05 (m)	2,3,5,6		
**4'**		1.46 (m)		2,6	1.55 (m)	2,6		
**5**		4.23 (t 7.7)		6	4.31 (t 7.8)	2,3,4,6		
**6**		-		-	-	-		
**7**		5.33 (dd 4.4, 2.4)		-	5.28 (dd 5.3, 2.3)	6,9,12,16	3.95 (t 3.5)	9,13,15
**8**		2.82(br.d 20.8)		-	2.67 (br. d 19.1)	16	2.52 (br. d 20.1)	
**8'**		2.55 (m)		9,10,16	2.37 (m)	7,9,10,16	2.31 (m)	7,9,10,16
**9**		-		-	-	-	-	-
**10**		5.98 (d 7.4)		8,12,18	5.56 (m)	8,11,12,18	5.87 (d 7.3)	8,12,18
**11**		6.24 (d 7.4)		9,13,16	2.12 (m)	-	6.08 (d 7.4)	9,13,16
**11'**		-		-	2.52 (m)	18	-	-
**12**		-		-	3.31 (m)	10,11,14	-	-
**13**		-		-	-	-	-	-
**14**		6.03 (s)		12,13,15,16,19	5.81 (q 1.8)	12,13,15,16,19	5.93 (s)	12,13,15,16,19
**15**		-		-	-	-	-	-
**16**		-		-	-	-	-	-
**17**		0.96 (s)		7,12,15,16	0.96 (s)	7,12,15,16	0.79 (s)	7,12,15,16
**18**		1.85 (s)		8,9,10	1.68 (s)	8,9,10	1.82 (s)	8, 9,10
**19**		2.55 (m)		13,20	2.39 (m)	13,14,20	2.48 (m)	12,13,14,20
**20**		1.16 (t 7.4)		13,19	1.11 (t 7.3)	13,19	1.14 (t 7.4)	13,19
**-OH**		-		-	-	-	4.43 (s)	-

The NMR data for aculene A were very comparable to those of aculene B (Table 1 and Table 2), yet differences were observed corresponding to the additional double bond in aculene A. The chemical shift of H-10 and H-14 is seen to differ slightly and furthermore an additional resonance was present in the ^1^H-NMR spectrum in the aromatic/alkene area for compound A. This was located at a chemical shift of δ_H_ = 6.24 ppm and assigned H-11. The only other location where the spectrum differed significantly was due to the extra diastereotopic CH_2_ groups in the 7-membered ring of aculene B. For this, and the diastereotopic CH_2_ group H8/H8' present in both compounds, rather large coupling constants were observed due to the geminal coupling. In compound A the H-8 doublet had a coupling constant of 20.8 Hz. Another notable chemical shift is the carbon chemical shift of C-13. This was at δ_C_ = 175.3 and 183.8 ppm, respectively for the two compounds, which is rather far downfield.

**Table 2:** ^13^C-NMR spectroscopic data (125 and 200 MHz, DMSO-*d_6_*) for aculenes A–C.

Position	Aculene A	Aculene B	Aculene C
	δ_C_	δ_C_	δ_C_
**1**	-	-	-
**2**	45.1	44.7	-
**3**	22.3	22.3	-
**4**	27.6	27.6	-
**5**	58.4	58.3	-
**6**	168.0	167.8	-
**7**	72.9	73.5	67.6
**8**	36.5	35.5	40.2
**9**	141.7	130.7	142.5
**10**	120.0	122.7	119.7
**11**	118.5	24.9	117.8
**12**	142.5	44.6	144.3
**13**	175.3	183.8	174.3
**14**	125.3	122.8	125.6
**15**	205.8	208.2	208.2
**16**	51.7	54.4	53.6
**17**	17.9	17.1	18.3
**18**	26.9	28.3	27.1
**19**	20.1	23.1	19.8
**20**	12.0	10.8	11.8

A third and related compound (aculene C) was also characterized. The UV spectrum was similar to the UV spectrum observed for aculene A (Figure 3) and the molecular formula was determined by HRMS to be C_14_H_18_O_2_. The NMR data was comparable to that of aculene A (Table 1 and Table 2), though with an absence of resonances originating from the proline part. Elucidation of the structure revealed that the compound was related to the aculenes, therefore it was named aculene C (Figure 4). Aculene C is a likely precursor to aculene A, having the same carbon skeleton, but missing the proline moiety. Analysis of the UHPLC-DAD-HRMS data showed a fourth compound, eluting close by aculene C, present in minute amounts, with a mass difference of 2 Da compared to aculene C, and with a UV spectrum very similar to aculene B (Figure 3). The HRMS data displayed the same adducts and fragmentation pattern as observed for aculene C. This compound, aculene D, is believed to have a similar structure as aculene C, but with the absence of the double bond between C-11 and C-12, as seen with the relation between aculenes A and B. This structure has not been verified by NMR experiments.

The stereochemistry of aculenes A-C was elucidated by NOEs, *J*-couplings and Marfey’s reaction [34], from which it was determined that aculenes A and B contained L-proline. The stereocenters (C-5, C-7 and C-16) of aculenes A and B could not be connected, due to lack of NOEs from the proline to the bicyclic system and the free rotation of proline. The relative stereochemistry of stereocenters C-7 and C-16, as well as C-12 for aculene B, is depicted in Figure 4, suggested by NOEs and backcalculated ^3^*J*-couplings of H-7 (see Supplementary Figure S37 and Supplementary Tables S1 and S2). Relevant NOEs are depicted for aculene B in Figure 5.

We hypothesize the biosynthesis of the aculenes to originate from a terpene pathway. Aculene C and D consist of fourteen carbons, which could originate from a sesquiterpene with the loss of one carbon atom, possibly at C9. Aculene A and B furthermore contain the amino acid proline.

**Figure 6:**
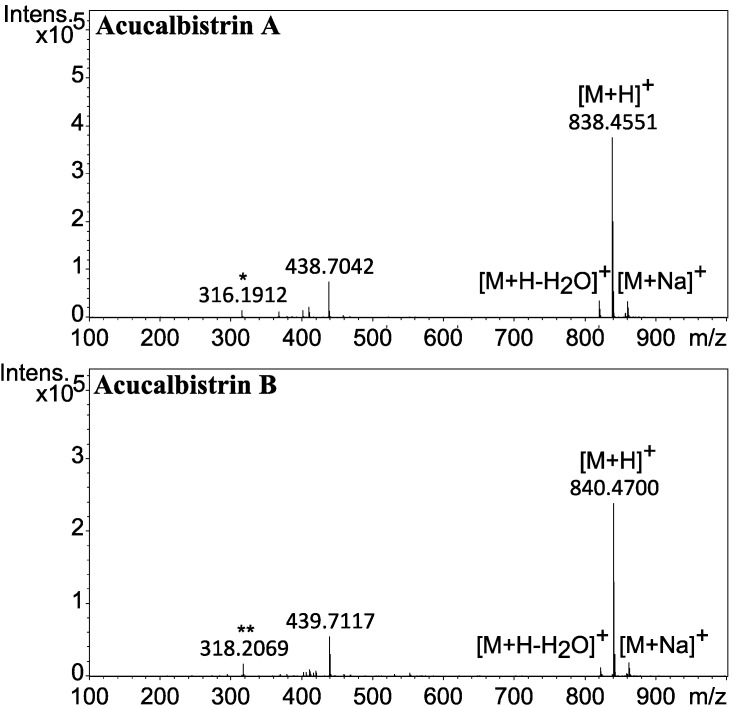
Mass spectra of acucalbistrin A (top) and acucalbistrin B (bottom). A fragment corresponding to *m/z* of the molecular ion [M+H]^+^ of aculene A (*) and aculene B (**) are observed for acucalbistrin A and B respectively.

The two compounds marked as acucalbistrin A and B in Figure 1 were also targeted. These compounds were intriguing because they could not be dereplicated, and because the HRMS data suggested that the size were somewhat larger than typical secondary metabolites. The molecular formulas for the two compounds were determined to be C_50_H_63_NO_10_ and C_50_H_65_NO_10_, the mass spectra are depicted in Figure 6.

A feature noted in both mass spectra was a fragment corresponding to the mass of aculenes A and aculene B, respectively. Different chromatographic approaches were taken to purify both compounds, which however proved challenging as the compounds were unstable even under mild conditions. Apparently acucalbistrin A was degrading to aculene A and another compound X, while acucalbistrin B was degrading to aculene B and the same compound X. Analysis by UHPLC-DAD-HRMS of a small semipreparative proportion of this degradation product revealed to our surprise this compound to be calbistrin A. This was verified by the UHPLC-DAD-HRMS data by comparison to the known compounds, see Supplementary Figure S39.

The combined mass of aculene A and calbistrin A is higher than that of acucalbistrin A (and the same for the case of acucalbistrin B), where the difference corresponds to a single water molecule. We therefore hypothezise that the acucalbistrins undergo hydrolysis and with knowledge on the structures of aculenes A–B and calbistrin A, a possible structure could therefore be an amide bond between the nitrogen in the proline moiety in aculene to the carbonyl in the carboxylic acid part of calbistrin A (see Supplementary Figure S38). This would account for the observed masses. These acucalbistrin structures have not been verified by NMR spectroscopy. We note that the UV spectra of acucalbistrin A and B have UV_max_ values comparable to those observed for calbistrin A (Figure 3). The slight shift observed may be due to the aculene moiety being attached directly to the carboxylic acid, which is part of the conjugated system in calbistrin A. The proposed structures are given in Supplementary Figure S38.

### 2.2. Discovery of Novel Indole Terpenoids

**Figure 7:**
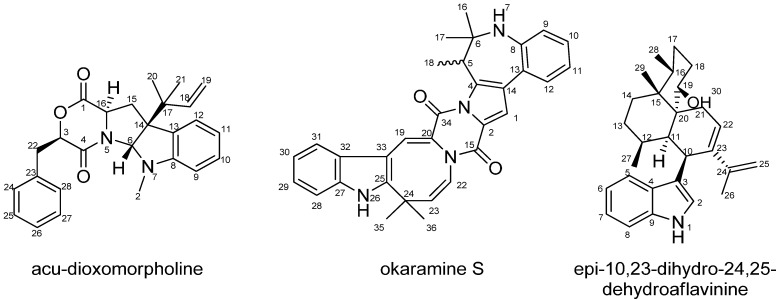
The structure of acu-dioxomorpholine, okaramine S and epi-10,23-dihydro-24,25-dehydroaflavinine.

Three other compounds were isolated and structure elucidated resulting in the novel compounds acu-dioxomorpholine, okaramine S and epi-10,23-dihydro-24,25-dehydroaflavinine with the structures shown in Figure 7.

NMR data for the compounds are presented in Table 3, Table 4, and Table 5, respectively. The structure of acu-dioxomorpholine reminds one of a diketopiperazine, but this structure differs in that it contains a lactone. The chemical shift of the proton adjacent to the lactone oxygen (H-3) clearly differs compared to the chemical shift of a proton in ordinary diketopiperazines, which is typically reported to be around δ_H_ = 3.50–4.50 ppm [35,36]. For acu-dioxomorpholine the proton chemical shift is δ_H_ = 5.32 ppm. The carbon chemical shift of C-3 is also shifted significantly downfield, with δ_C_ = 78.1 ppm for the acu-dioxomorpholine, compared to around 60 ppm for diketopiperazines. Two compounds have been reported by Wang *et al.* with the same lactone feature [37], but differ by having leucine incorporated instead of phenylalanine and by lacking the *N*-methylation. Otherwise the NMR data are comparable.

NOESY experiments enabled determination of the relative stereochemistry of acu-dioxomorpholine. NOE connectivities were found between H-3 and H-16 placing these protons on the same side of the ring system. These had no NOE connectivities to H-6, which however displayed NOE connectivities to H-22 as well as several of the protons in the prenyl moiety (H-18, H-19, H-19' and H-20), strongly indicating the positioning of the prenyl group and H-6 on the other side of the ring system as compared to H-3 and H-16.

**Table 3:** NMR spectroscopic data (500 and 800 MHz, DMSO-*d_6_*) for acu-dioxomorpholine.

Position	δ_H_ (*J* in Hz)	δ_C_	HMBC	Noesy
1	-	168.0	-	-
2	2.96 (3H, s)	32.7	6, 8	6,9
3	5.32 (1H, dd, 8.8, 3.4)	78.1	4, 22, 23	16, 22, 22', 24/28
4	-	163.5	-	-
5	-	-	-	-
6	5.43 (1H, s)	81.9	2, 4, 8, 13, 14, 15, 16, 17	12, 18, 19, 19', 20, 22
7	-	-	-	-
8	-	150.8	-	-
9	6.44 (1H, d, 7.6)	105.6	11, 13	2, 10
10	7.09 (1H, td, 7.6, 0.8)	128.5	8, 12	9, 11
11	6.64 (1H, t, 7.3)	116.9	9, 12, 13	10, 12
12	7.17 (1H, d, 7.3)	124.1	8, 10, 14	11, 15, 18, 20, 21
13	-	128.8	-	-
14	-	59.6	-	-
15	2.43 (1H, dd, 12.7, 6.6)	36.6	1, 6, 13, 14, 16	12, 15', 16
15'	2.21 ( 1H, dd, 12.7, 11.1)	36.6	1, 13, 14, 16	15, 16, 18, 20, 21
16	4.17 (1H, dd, 11.1, 6.6)	56.7	1, 15	3, 15, 15'
17	-	40.1	-	-
18	5.87 (1H, dd, 17.4, 11.0)	143.3	14, 17, 21	6, 12, 15', 19, 19', 20, 21
19	5.02 (1H, dd, 17.4, 1.1)	113.7	14, 17, 18	6, 18, 19', 20, 21
19'	5.06 (1H, dd, 11.0, 1.1)	113.6	14, 17, 18	6, 18, 19, 20, 21
20	0.82 (3H, s)	22.4	14, 17, 18, 21	6, 12, 15', 18, 19, 19', 21
21	0.97 (3H, s)	21.7	14, 17, 18, 20	12, 15', 18, 19, 19', 20
22	2.98 (1H, dd, 14.8, 8.8)	34.9	3, 4, 23, 28	3, 6, 22', 24/28
22'	3.33 (1H, m)	34.9	3, 4, 23, 28	3, 22, 24/28
23	-	136.3	-	-
24	7.26 (1H, m)	129.2	22, 26, 28	3, 22, 22'
25	7.28 (1H, m)	127.7	-	26
26	7.22 (1H, tt, 7.2, 1.6)	126.2	28	25
27	7.28 (1H, m)	127.7	23, 25	-
28	7.26 (1H, m)	129.2	-	3, 22, 22'

**Table 4:** NMR spectroscopic data (500 and 800 MHz, DMSO-*d_6_*) for okaramine S.

Position	δ_H_ (*J* in Hz)	δ_C_	Hmbc	Noesy
1	7.64 (1H, s)	114.7	2, 4, 14, 15	-
2	-	123.8	-	-
4	-	137.6	-	-
5	4.26 (1H, q, 6.5)	43.2	4, 14, 18	18
6	-	51.2	-	-
7	5.87 (1H, s)	-	5, 9, 13, 17	9
8	-	144.5	-	-
9	6.90 (1H, d, 7.6)	118.8	11, 13	10
10	6.99 (1H, t, 7.6)	127.4	8, 12	9
11	6.65 (1H, t, 7.6)	116.8	9, 13	12
12	7.74 (1H, d, 7.6)	128.3	8, 10, 14	1,11
13	-	115.3	-	-
14	-	124.9	-	-
15	-	153.4	-	-
16	0.95 (3H, s)	27.1	5, 6, 17	17
17	1.39 (3H, s)	29.3	5, 6, 16	16
18	1.18 (3H, d, 7.2)	18.1	4, 5, 6	-
19	7.76 (1H, s)	117.5	20, 25, 32, 33, 34	-
20	-	125.2	-	-
22	5.90 (1H, d, 8.3)	123.6	23, 24	23
23	6.15 (1H, d, 8.3)	140.2	22, 24, 35/36	22
24	-	35.8	-	-
25	-	149.8	-	-
26	11.70 (1H, s)	-	25, 32, 33	-
27	-	134.2	-	-
28	7.44 (1H, m)	112.3	-	29/30
29	7.17 (1H, m)	121.7	31	28, 31
30	7.17 (1H, m)	121.7	31	28, 31
31	7.63 (1H, m)	116.1	27, 29/30	12, 29/30
32	-	105.3	-	-
33	-	129.5	-	-
34	-	157.6	-	-
35	1.69 (3H, s)	26.7	23, 24, 25, 35/36	-
36	1.69 (3H, s)	26.7	23, 24, 25, 35/36	-

**Figure 8:**
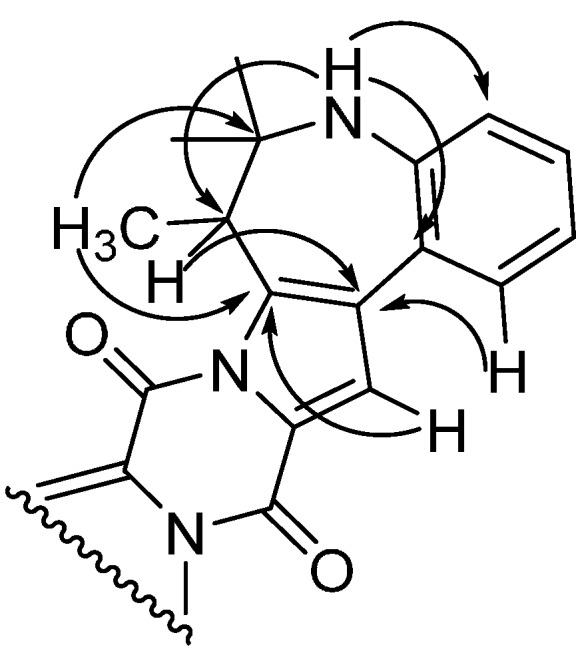
Key HMBCs substantiating the seven-membered ring in okaramine S.

For okaramine S the molecular formula was by HRMS predicted to be C_32_H_30_N_4_O_2_. The azocinoindole part of the novel okaramine (Figure 7) is identical to that observed in okaramines A and H (Scheme 1), also produced by *A. aculeatus* [12]. The remaining part is highly similar, also originating from tryptophan and an isoprene unit, but in okaramine S, the terpene is fused with the ring system of the tryptophan moiety resulting in an intriguing, additional seven-membered ring, not previously reported in any okaramine. Key HMBCs from this part of okaramine S are depicted in Figure 8.

**Table 5:** NMR spectroscopic data (500 MHz, DMSO-*d_6_*) for epi-10,23-dihydro-24,25-dehydroaflavinine.

Position	δ_H_ (*J* in Hz)	δ_C_	HMBC	H2BC	NOESY
1	10.66 (s)	-	2, 3, 4, 9	2	2,8
2	7.07 (d 2.0)	122.7	3, 4, 9	-	1, 23, 25, 27
3	-	114.4	-	-	
4	-	126.7	-	-	
5	7.38 (d 7.8)	116.8	7, 9	6	10, 11, 18
6	6.96 (t 7.2)	117.7	4, 8	5, 7	
7	7.02 (t 7.3)	120.0	5, 9	6, 8	
8	7.29 (d 8.0)	110.9	4, 6	7	1
9	-	135.5	-	-	
10	3.59 (dd 13.3, 5.0)	33.4	2, 3, 11, 12, 23, 24	11, 23	5, 11, 19, 25, 26
11	2.47 (m)	37.6	10, 12, 23	10, 12	5, 10, 12, 13, 16, 19
12	1.27 (m)	28.9	-	17	11
13	1.53 (m)	28.1	29	-	11, 13', 16
13'	0.81 (d 12.9)	28.1	-	-	13
14	1.46 (m)	27.4	-	-	
14'	1.08 (d 13.7)	27.4	27	-	28
15	-	38.2	-	-	
16	2.04 (m)	30.4	-	17, 28	11, 13, 17', 28
17	1.71 (m)	24.8	-	18	
17'	1.22 (m)	24.8	-	-	
18	1.99 (d 11.3)	29.6	-	-	5, 19
18'	1.74 (m)	29.6	-	-	19, 30
19	4.64 (s)	65.6	-	-	10, 11, 18, 18', 22, 26
20	-	42.9	-	-	
21	2.07 (m)	23.5	-	-	29, 30
21'	1.65 (m)	23.5	19, 23	-	27
22	1.85 (m)	26.6	20, 24	21, 23	19, 30
22'	1.54 (m)	26.6	-	-	
23	3.13 (m)	42.3	24, 25, 26	10, 22	2, 25, 27
24	-	149.8	-	-	
25	4.81 (d 1.8)	110.4	23, 26	26	2, 10, 23, 25'
25'	4.58 (d 1.8)	110.4	23, 26	26	25, 26
26	1.45 (s)	17.7	23, 24, 25	-	10, 19, 25'
27	1.21 (s)	21.1	11, 12	12	2, 21', 23
28	0.71 (d 6.7)	15.4	15, 16, 17	16	14', 16, 17'
29	0.92 (s)	17.7	13, 15, 16, 20	-	17, 21, 30
30	4.28 (d 4.3)	-	19, 20	19	18', 21, 22, 29

The molecular formula of the aflavinine analogue was predicted by HRMS to be C_28_H_39_NO and dereplication indicated it could be aflavinine or an analogue. Since no aflavinine or analogue was previously described in *A. aculeatus*, this compound was also a target for isolation and structural elucidation. The NMR data, see Table 5, suggested the structure of 10,23-dihydro-24,25-dehydroaflavinine.

10,23-Dihydro-24,25-dehydroaflavinine has earlier been reported from sclerotia from *A. flavus*, *A. parasiticus* and *A. tubingensis* [38]. The compound has not been reported from *A. aculeatus*, but it is known to be produced by the related black *Aspergillus A. costaricaensis* [39]. The stereochemistry of epi-10,23-dihydro-24,25-dehydroaflavinine was solved by the use of NOEs and *J*-couplings. The relative stereochemistry was found to be equal to that of 10,23-dihydro-24,25-dehydroaflavinine [38]. For investigation of the absolute configuration of the purified compound, optical rotation was measured. Two different values were obtained, [α] = 63.58° and [α] = 9.08° in MeOH and CHCl_3_ respectively. These values were compared to the value of [α]_D_ = −1.20°, which has earlier been reported for 10,23-dihydro-24,25-dehydroaflavinine in CHCl_3_ [38]. Due to those opposite signs, our result suggests that the compound from *A. aculeatus* is the enantiomer of 10,23-dihydro-24,25-dehydroaflavinine, wherefore we have named it epi-10,23-dihydro-24,25-dehydroaflavinine. The 3D structure (see Supplementary Figure S40) was suggested by the isolated spin pair approximation [40].

### 2.3. Biological Testing of the Novel A. aculeatus Metabolites

Aculenes A–C, acu-dioxomorpholine, okaramine S and epi-10,23-dihydro-24,25-dehydroaflavinine were tested for antifungal activity against *Candida albicans*. Endpoint optical density from compound screens were normalized with the negative controls and susceptibility evaluated as percentage reduction in optical density. None of the compounds showed significant antifungal activity.

### 2.4. Production of Sclerotia Reveals a Highly Regulated Metabolic Profile

**Figure 9:**
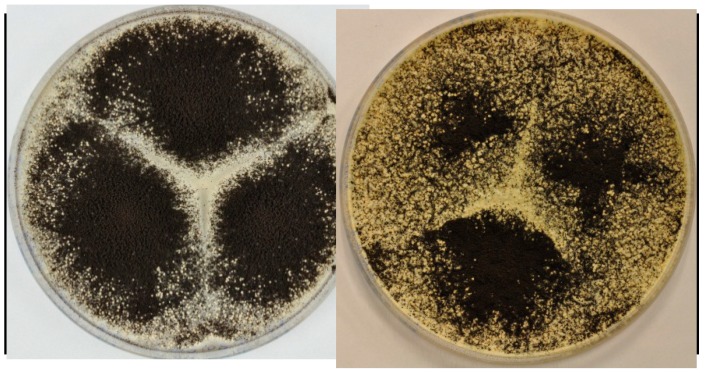
The figure shows production of sclerotia on YES (left) and CYA media (right) after growth for 7 days at 25 °C in the dark. The sclerotia are the white to cream-colored structures seen among the black conidial *Aspergillus* heads.

It has recently been demonstrated that sclerotium production can be prompted under specific conditions for a number of the black aspergilla [41]. This study showed that some metabolites are highly upregulated inside the sclerotia, compared to the metabolites in the mycelium. *A. aculeatus* is capable of producing sclerotia in vast amounts. Production was demonstrated on CYA and YES media, see Figure 9.

**Figure 10:**
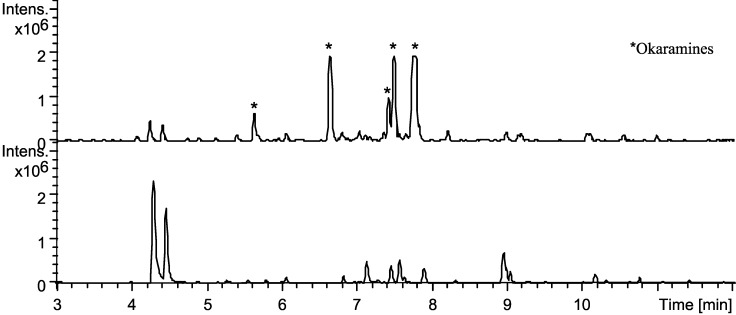
Base peak chromatograms of extraction from sclerotia (top) and plug extractions of *A. aculeatus* IBT 21030 after cultivation on YES agar for 7 days at 25 °C in the dark (bottom) for reference. The chromatograms are to scale. Stars indicate okaramines produced in the sclerotium extract.

Analysis of extracts of sclerotia from MM, CYA and YES showed large upregulations of okaramines, see Figure 10. Aculenes A–C were still produced, though in smaller amounts. Furthermore the analysis showed that production of secalonic acids abolished on MM and CYA. The calbistrins were still produced and the same were acucalbistrins A–B, though in trace amounts. Some okaramine analogues have shown activity against silkworms [42,43,44] and the substantial upregulation of okaramines can be connected to sclerotium production as a defense mechanism to protect against insects. Sclerotia are survival structures and the production is often provoked by extreme conditions [41,45].

## 3. Experimental Section

### 3.1. Fungal Growth, Strains and Media

For standard screening the fungal strain was inoculated as three point stabs on solid media and incubated for seven days at 25 °C. Media was prepared as described by Samson *et al.* [25] The strain IBT 21030 was compared to the culture ex type IBT 3244 = CBS 172.66 = ATCC 16872 = IMI 211388 = WB 5094, in order to authenticate the identity of the isolate.

### 3.2. Large Scale Extraction

For the large scale preparation, *A. aculeatus* (IBT 21030) was inoculated as three point stabs on 200 plates of solid YES medium, and incubated in the dark for 7 days at 25 °C. The fungi were harvested and extracted. Ten Petri plates of fungi were transferred to separate stomacher bags. To each bag 100 mL of ethyl acetate (EtOAc) containing 1% formic acid (FA) were added and the bags were subsequently crushed using a stomacher for 30–60 s. After one hour the liquid phase was filtered and concentrated *in vacuo*. The stomacher bags still containing the fungi were again filled with EtOAc containing 1% FA, and then left overnight. The following day the solution was filtered and then concentrated *in vacuo*. The combined extract was dissolved in 800 mL methanol (MeOH) and H_2_O purified and deionized by a Millipore system through a 0.22 μm membrane filter (MQ H_2_O) (9:1) and 800 mL of heptane added, whereafter the phases were separated. To the MeOH/MQ H_2_O phase 800 mL MQ H_2_O was added, and metabolites were then extracted with 4 × 400 mL dichloromethane (DCM). The phases were then concentrated separately *in vacuo.*

### 3.3. Plug Extraction

For standard screening three plugs were taken from one colony by use of a 6 mm plug drill; one from the center of the colony, one from the edge near the other colonies and one from the edge as far away from the other colonies. The plugs were transferred to 2 mL vials and 500 µL extraction solvent were added. A mixture of MeOH/DCM/EtOAc (1:2:3 v/v/v) containing 1% FA was used. To each sample 40 μL chloramphenicol in ethanol (500 μg/mL) was added as an internal standard. The vials were placed in an ultrasonic bath (Branson 2510 or 3520) for 60 min. The extract was then transferred to a clean vial and evaporated to dryness. This was either achieved by leaving the vials in a fume hood over night or by applying nitrogen airflow at 25–32 °C. After evaporation 500 µL MeOH was added and the sample was then ultrasonicated for 20 minutes. The extract was then filtered using a 0.45 μm PTFE filter.

### 3.4. Sclerotium Extraction

*A. aculeatus* IBT 21030 was three-point inoculated on YES, CYA and MM plates and incubated at 30 °C for ten days and then at room temperature for eight days. Sclerotia were harvested by applying MQ H_2_O to the plate and carefully harvest with Drigalski spatula. The liquid including the sclerotia and spores was filtred through sterilised Miracloth and washed with MQ H_2_O, allowing for the spores to pass through, while sclerotia were staying in the filter. Sclerotia were transferred to true Eppendorf tubes and washed with water to remove remaining spores. After evaporation of excess MQ H_2_O, two big and two small steel balls was added to each tube and the tubes were shaken at 2000 rpm for 2 × 60 s. To each tube 40 µL chloramphenicol (internal standard) and 1 mL MeOH/DCM/EtOAc (1:2:3 v/v/v) containing 1% FA was added and the extract was transferred to clean 2 mL vials. The samples were ultrasonicated for 1 h and the extract was transferred to clean vials and solvent was evaporated by applying airflow at 25–32 °C. The samples were redissolved in 500 µL MeOH, ultrasonicated for 20 min and filtrated to fresh vials using 0.45 μm PTFE filters.

### 3.5. UHPLC-DAD-HRMS Analysis

Analysis was performed using ultra-high-performance liquid chromatography (UPHLC) UV/Vis diode array detector (DAD) high-resolution MS (TOFMS) on a maXis 3G orthogonal acceleration quadrupole time of flight mass spectrometer (Bruker Daltonics, Bremen, Germany) equipped with an electrospray ionization (ESI) source and connected to an Ultimate 3000 UHPLC system (Dionex, Sunnyvale, CA, USA). The column used was a reverse-phase Kinetex 2.6-μm C_18_, 100 × 2.1 mm (Phenomenex, Torrance, CA, USA), and the column temperature was maintained at 40 °C. A linear water-acetonitrile (ACN) (LCMS-grade) gradient was used (both solvents were buffered with 20 mM FA) starting from 10% (vol/vol) ACN and increased to 100% in 10 min, maintaining this rate for 3 min before returning to the starting conditions in 0.1 min and staying there for 2.4 min before the following run. A flow rate of 0.4 mL·min^−1^ was used. TOFMS was performed in ESI+ with a data acquisition range of 10 scans per second at *m/z* 100–1000. The TOFMS was calibrated using Bruker Daltonics high precision calibration algorithm (HPC) by means of the use of the internal standard sodium formate, which was automatically infused before each run. UV/VIS spectra were collected at wavelengths from 200 to 700 nm. Data processing was performed using DataAnalysis 4.0 software (Bruker Daltonics).

### 3.6. NMR

The 1D and 2D spectra were recorded on a Varian Unity Inova-500 MHz spectrometer located at DTU, or on a Bruker Avance 800 MHz spectrometer located at the Danish Instrument Centre for NMR Spectroscopy of Biological Macromolecules at Carlsberg Laboratory. Spectra were acquired using standard pulse sequences. The deuterated solvent was DMSO-*d_6_* and signals were referenced by solvent signals for DMSO-*d_6_* at δ_H_ = 2.49 ppm and δ_C_ = 39.5 ppm. The NMR data was processed in MestReNova V.6.0.1–5391 or Bruker Topspin. Chemical shifts are in ppm (*δ*) and scalar couplings are reported in hertz (Hz). The sizes of the *J* coupling constants reported in the tables are the experimentally measured values from the 1D ^1^H and DQF-COSY spectra. There are minor variations in the measurements which may be explained by the uncertainty of *J* and the spectral digital resolution.

Distances for the epi-10,23-dihydro-24,25-dehydroaflavinine were obtained from 2D NOESY experiments using the isolated spin pair approximation (ISPA) [40]. The linear range was increased by the method suggested by Macura *et al.* [46,47]. The used mixing time was 150 ms. Different mixing times were used to construct a buildup curve to ensure that only crosspeaks which fitted the ISPA were used.

The ^3^*J*-couplings from angles in the 3D structures of epi-10,23-dihydro-24,25-dehydroaflavinine and the aculenes were calculated by the Haasnoot-DeLeeuw-Altona (HLA) equation and by DFT computations of the final structure [48,49].

The simulations were conducted using the program Maestro (Version 9.3.515, MMshare Version 2.1.515) from the Schrödinger suite. Conformational searches in implicit solvents (DMSO) were run by MacroModel (version 9.9, Schrödinger, LLC, New York, NY, USA, 2012) using the force fields OPLS2005 and MMFFs. Monte Carlo torsional sampling was used to generate the structures and the minimization method was PRCG. The number of steps were 20,000, and only conformations within 30 kJ/mol of the found minimum were considered. The solvent DMSO was treated as a constant dielectric constant of 47.0. Both force fields gave similar results.

Selected structures were further optimized by HF/3-21G using Jaguar (Jaguar, version 7.9, 2012, Schrödinger, LLC, New York, NY, USA,) followed by DFT (for epi-10,23-dihydro-24,25-dehydroaflavinine) [50]. DFT calculations were carried out by Gaussian09 [51] using cam-B3LYP/6-311++G(d,p) for optimization and B3LYP/6-31G(d,p) u+1s for calculating *J*-coupling constants (only Fermi Contact contribution considered, see reference [49]).

### 3.7. Marfey’s Reaction

Aculenes A and B (100 µg) were hydrolyzed with 6 M HCl (200 µL) in 2 mL analytical vials with lids, and were left at 110 °C for 22 h. The solvent was removed at a speedvac. For reference, 50 µL (2.5 µmol) standard l- and d-proline were prepared. To all vials 50 µL H_2_O, 20 µL 1 M NaHCO_3_ and 100 µL 1% 1-fluoro-2,4-dinitrophenyl-5-L-alanineamide (FDAA) in acetone were added. The vials were left for 40 °C for 1 h. 10 µL 2 M HCl was added followed by 820 µL MeOH and HPLC-DAD-MS data were acquired.

### 3.8. Purification of Metabolites

The DCM phase from the large scale extraction consisting of 2.375 g was absorbed onto Sepra ZT C18 (Phenomenex) and dried before packing into a 50 g (~66 mL) SNAP column (Biotage, Uppsala, Sweden) with Septra ZT C18 material. The extract was then fractionated using an Isolera flash purification system (Biotage). The gradient started with 15:100 MeOH/H_2_O in the first three column volumes (CV), then went to 100% MeOH over 18 CVs and stayed here for 3 CVs using a flow rate of 40 mL/min. MeOH was of HPLC grade and H_2_O was purified and deionized by Millipore system through 0.22 μm membrane filter (MQ H_2_O) and both were added 50 ppm trifluoroacetic acid (TFA). Fractions were automatically collected 1 CV at a time. The fractions were subjected to further purification on a semi-preparative HPLC, which was either a Waters 600 Controller with a 996 photodiode array detector (Waters, Milford, MA, USA) or a Gilson 322 Controller connected to a 215 Liquid Handler, 819 Injection Module and a 172 DAD (Gilson, Middleton, WI, USA). This was achieved using a Luna II C18 column (250 × 10 mm, 5 μm, Phenomenex). 50 ppm TFA was added to ACN of HPLC grade and MQ H_2_O. For choice of system, flow rate, gradients and yields see descriptions for the specific compound.

*Aculenes A and B.* The three first Isolera flash chromatography fractions, eluding with 10%–25% MeOH, were subjected to further purification on the waters semi-preparative HPLC. A linear water-ACN gradient was used starting with 15% ACN and increasing to 100% over twenty minutes using a flow rate of 4 mL/min. Two compounds eluting with 54% and 56% ACN respectively, were collected. This yielded two pure compounds, 3.0 mg of aculene A and 3.7 mg of aculene B.

*Aculene A*: HRMS: *m/z* = 316.1910. [M+H]^+^, calculated for [C_19_H_25_NO_3_+H]^+^: *m/z* = 316.1907. 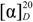 = +0.63° (MeOH). ^1^H- and ^13^C-NMR (see Table 1 and Table 2).

*Aculene B*: HRMS: *m/z* = 318.2068. [M+H]^+^, calculated for [C_19_H_27_NO_3_+H]^+^: *m/z* = 318.2064. 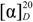 = +6.96° (MeOH). ^1^H- and ^13^C-NMR (see Table 1 and Table 2).

*Aculene C*. One of the Isolera flash chromatography fractions, eluding with 50% MeOH, was subjected to further purification on the waters semi-preparative HPLC. An isocratic water-ACN gradient was used starting with 40% ACN over twenty minutes using a flow rate of 4 mL/min. A compound eluding with 40% ACN was collected. This yielded 2.0 mg of aculene C.

HRMS: *m/z* = 319.1382 [M+H]^+^, calculated for [C_14_H_18_O_2_+H]^+^: *m/z* = 319.1379. 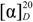 = 0.00° (MeOH). ^1^H- and ^13^C-NMR (see Table 1 and Table 2).

*Calbistrin A and C*. One of the Isolera flash chromatography fractions, eluding with 100% MeOH, was subjected to further purification on the waters semi-preparative HPLC. An isocratic water-ACN method was used with 52% ACN over eighteen minutes using a flow rate of 4 mL/min. Two compounds eluding at 52% ACN were collected. This yielded 5.2 mg of calbistrin A and 4.6 mg of calbistrin C.

*Calbistrin A*: HRMS: *m/z* = 563.2622 [M+Na]^+^, calculated for [C_31_H_40_O_8_+Na]^+^: *m/z* = 563.2615. 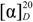 = +46.1° (MeOH). ^1^H-NMR (499.87 MHz, DMSO-*d_6_*, 25 °C, 2.49 ppm): 0.77 (3H, d, *J* = 7.1 Hz), 0.97 (3H, d, *J* = 7.1 Hz), 1.13 (3H, s), 1.22 (3H, s), 1.32 (1H, m), 1.69 (3H, s), 1.98 (3H, s), 2.02 (1H, m), 2.30 (1H, dd, *J* = 13.8, 3.8 Hz), 2.37 (1H, m), 2.41 (1H, m), 2.69 (1H, dd, *J* = 13.8, 8.2 Hz), 2.80 (1H, m), 3.94 (1H, d, *J* = 9.4 Hz), 5.06 (1H, m, -OH), 5.10 (1H, m), 5.59 (1H, d, *J* = 9.6 Hz), 5.70 (1H, s), 5.86 (1H, m), 5.88 (1H, d, *J* = 14.9 Hz), 5.97 (1H, d, *J* = 9.8 Hz), 6.05 (1H, d, *J* = 11.2 Hz), 6.30 (1H, d, *J* = 12.0 Hz), 6.38 (1H, d, *J* = 15.1 Hz), 6.68 (1H, dd, *J* = 15.1, 11.2 Hz), 7.51 (1H, dd, *J* = 14.9, 12.0 Hz; ^13^C-NMR (125.70 MHz, DMSO-*d_6_*, 25 °C, 39.5 ppm): 10.9, 12.3, 13.0, 13.7, 20.6, 25.4, 26.0, 34.7, 39.8, 43.6, 43.7, 56.2, 56.3, 68.3, 78.3, 121.2, 126.4, 127.0, 127.2, 130.8, 132.7, 133.8, 135.7, 139.4, 140.9, 143.4, 173.9, 212.8.

*Calbistrin C:* HRMS: *m/z* = 565.2776 [M+Na]^+^, calculated for [C_31_H_42_O_8_+Na]^+^: *m/z* = 565.2772. 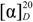 = +8.67° (MeOH); ^1^H-NMR (499.87 MHz, DMSO-*d_6_*, 25 °C, 2.49 ppm): 0.77 (3H, d, *J* = 7.1 Hz), 0.95 (3H, d, *J* = 7.1 Hz), 1.01 (3H, s), 1.21 (1H, m), 1.27 (3H, s), 1.71 (3H, s), 1.93 (1H, m), 1.98 (3H, s), 2.35 (1H, m), 2.38 (1H, qin, *J* = 8.4), 2.56 (1H, m), 2.92 (1H, dt, *J* = 17.5, 7.5 Hz), 2.99 (1H, m), 3.56 (2H, m), 3.95 (1H, dd, *J* = 9.4, 3.1 Hz), 5.09 (1H, br.d, *J* = 3.5 Hz, -OH), 5.05 (1H, s, -OH), 5.20 (1H, m), 5.37 (1H, d, *J* = 9.9 Hz), 5.59 (1H, m), 5.88 (1H, d, *J* = 14.9 Hz), 5.90 (1H, d, *J* = 9.9 Hz), 6.07 (1H, d, *J* = 11.1 Hz), 6.31 (1H, d, *J* = 12.0 Hz), 6.39 (1H, d, *J* = 15.1 Hz), 6.69 (1H, dd, *J* = 15.1, 11.1 Hz), 7.51 (1H, dd, *J* = 14.9, 12.0); ^13^C-NMR (125.70 MHz, DMSO-*d_6_*, 25 °C, 39.5 ppm): 10.9, 12.3, 13.0, 13.7, 20.6, 25.4, 26.0, 34.7, 39.8, 43.6, 43.7, 56.2, 56.3, 68.3, 78.3, 121.2, 126.4, 127.0, 127.2, 130.8, 132.7, 133.8, 135.7, 139.4, 140.9, 143.4, 173.9, 212.8.

*Acu-dioxomorpholine*. One of the Isolera flash chromatography fractions, eluding with 95% ACN, was subjected to further purification on the waters semi-preparative HPLC). A linear water-ACN gradient was used starting with 60% ACN increasing to 100% over twenty minutes using a flow rate of 4 mL/min. A compound eluding with 85% ACN was collected. This yielded 2.1 mg of acu-dioxomorpholine. HRMS: *m/z* = 417.2176 [M+H]^+^, calculated for [C_26_H_29_N_2_O_3_+H]^+^: *m/z* = 417.2173. 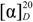 = −49.23° (MeOH). ^1^H- and ^13^C-NMR (see Table 3).

*Epi-10,23-dihydro-24,25-dehydroaflavinine*. One Isolera flash chromatography fraction eluding with 100% MeOH, was subjected to purification on the waters semi-preparative HPLC. A gradient of 15%–100% ACN over twenty minutes was used and the flow rate was 4 mL/min. A compound eluding with 100% ACN was collected. This yielded 8.7 mg of epi-10,23-dihydro-24,25-dehydroaflavinine. HRMS: *m/z* = 406.3113 [M+H]^+^, calculated for [C_28_H_39_NO+H]^+^: *m/z* = 406.3104. 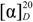 = +63.58° (MeOH); ^1^H- and ^13^C-NMR (see Table 5).

*Okaramine S*. One Isolera flash chromatography fraction, eluding with 100% MeOH, was subjected to purification on the Gilson semi-preparative HPLC. This was done using a gradient of 70%–100% ACN over fifteen minutes using a flow rate was 5 mL/min. A compound eluding with 80% ACN was collected. This yielded 1.7 mg of the pure compound. HRMS: *m/z* = 503.2447 [M+H]^+^, calculated for [C_32_H_30_N_4_O_2_+H]^+^: *m/z* = 503.2441. 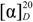 = −15.29° (MeOH); ^1^H- and ^13^C-NMR (see Table 4).

*Okaramine J*. One Isolera flash chromatography fraction, eluding with 74% MeOH, was subjected to purification on the waters semi-preparative HPLC. This was done using a gradient of 50%–100% ACN over twenty minutes using a flow rate of 4 mL/min. A compound eluding with 82% ACN was collected. This yielded 2.5 mg of the pure compound. HRESIMS: *m/z* = 525.2865 [M+H]^+^, calculated for [C_32_H_36_N_4_O_3_+H]^+^: *m/z* = 525.2860. 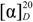 = +15.38° (MeOH); ^1^H-NMR (499.87 MHz, DMSO-*d_6_*, 25 °C, 2.49 ppm): 1.52 (6H, s, H-17, H-18), 1.68 (3H, s, H-34), 1.73 (3H, s, H-35), 1.86 (1H, m, H-20), 2.42 (1H, dd, 13.2, 6.8, H-20'), 2.99 (1H, dd, 15.2, 9.4, H-1), 3.14 (1H, dd, 16.3, 7.1, H-31), 3.23 (1H, dd, 16.8, 7.5, H-31'), 3.59 (1H, dd, 15.2, 4.2, H-1'), 4.46 (1H, dd, 9.1, 4.4, H-2), 4.67 (1H, dd, 11.3, 6.4, H-19), 5.05 (1H, dd, 10.5, 1.1, H-4), 5.08 (1H, dd, 17.4, 1.1, H-4'), 5.26 (1H, t, 7.4, H-32), 5.33 (1H, d, 4.5, H-29), 6.01 (1H, s , H-36), 6.12 (1H, d, 4.4, H-28), 6.22 (1H, dd, 17.4, 10.5, H-5), 6.32 (1H, s, H-3), 6.67 (1H, t, 7.5, H-24), 6.88 (1H, d, 7.5, H-25), 6.96 (1H, t, 7.5, H-12), 6.96 (H-26), 7.05 (2H, H-11, H-23), 7.35 (1H, s, H-10), 7.53 (1H, s, H-13), 10.67 (1H, s, H-8); ^13^C-NMR (125.70 MHz, DMSO-*d_6_*, 25 °C, 39.5 ppm): 17.5 (C-34), 24.7 (C-1), 25.2 (C-35), 27.6 (C-17,C-18), 28.2 (C-31), 39.0 (C-6), 40.8 (C-20), 55.1 (C-2), 58.2 (C-19), 83.5 (C-29), 85.5 (C-21), 104.3 (C-15), 110.7 (C-10), 110.9 (C-4), 117.6 (C-13), 118.4 (C-12, C-24), 118.9 (C-23), 120.0 (C-11), 121.5 (C-32), 122.7 (C-22), 128.3 (C-14), 131.0 (C-26), 132.0 (C-33), 134.5 (C-9), 141.1 (C-7), 145.8 (C-27), 146.0 (C-5), 167.5 (C-16), 169.7 (C-30).

### 3.9. Antifungal Susceptibility Testing

All compounds were screened for antifungal activity towards *Candida albicans* in accordance with the CLSI standards using RPMI-1640 medium adjusted to pH 7 with 0.165 M MOPS buffer [52]. The screening was performed as described by Holm *et al.* [53].

## 4. Conclusions

Investigation of the chemical profile of *Aspergillus aculeatus* by UHPLC-DAD-HRMS has identified several novel compounds some of which have been selected, purified and their structure elucidated by 1D and 2D NMR spectroscopy. A novel class of related compounds – the aculenes – has been discovered. Aculenes A and B are hybrids containing the amino acid proline but also a fourteen carbon moeity we hypothesize to originate from a sesquiterpene with the loss of one carbon atom. Aculene C is believed to be a precursor to aculene A, missing the proline part of the molecule. Moreover there is UHPLC-DAD-HRMS evidence suggesting a fourth structure, aculene D, a possible precursor to aculene B. Two other larger, however chemically unstable metabolites have been discovered. As they apparently were degrading to two known parts, aculene A and B respectively and calbistrin A, a tentative suggestion for their structures have been given. Two further novel compounds, acu-dioxomorpholine and okaramine S has also been discovered to be produced by *A. aculeatus*. Acu-dioxomorpholine has a remarkable structure, reminding of diketopiperazine, but with a lactone instead of the one lactame part and okaramine S displays a seven-membered ring, not previously seen in any reported okaramine. Furthermore the aflavinine analogue epi-10,23-dihydro-25,25-dehydroaflavinine was detected. Production of sclerotia was observed under specific conditions, and here an upregulation of okaramines was observed. Altogether the chemical profile of *Aspergillus aculeatus* has been examined and several novel compounds have been characterized.

## Supplementary Materials

^1^H, DQF-COSY, HSQC, HMBC, NOESY and UV spectra for the novel compounds as well as suggestion for the structures of acucalbistrin A and B and elucidation of the stereochemistry of aculenes A–D and epi-10,23-dihydro-24,25-dehydroaflavinine are available in the supplementary. Supplementary materials can be accessed at: http://www.mdpi.com/1420-3049/19/8/10898/s1.

## Supplementary Files

Supplementary File 1
