# Supplementary material for: Dereplication Guided Discovery of Secondary Metabolites of Mixed Biosynthetic Origin from Aspergillus aculeatus
Source: Molecules. 2014 Jul 25;19(8):10898–921. doi: 10.3390/molecules190810898 (PMC6271215; doi:10.3390/molecules190810898)
Supplement: Supplementary File 1 [file molecules-19-10898-s001.pdf]

Supplementary Materials

Figure S1. ^{1}H-NMR (500 MHz, DMSO-d_6 spectrum) of the new compound aculene A.

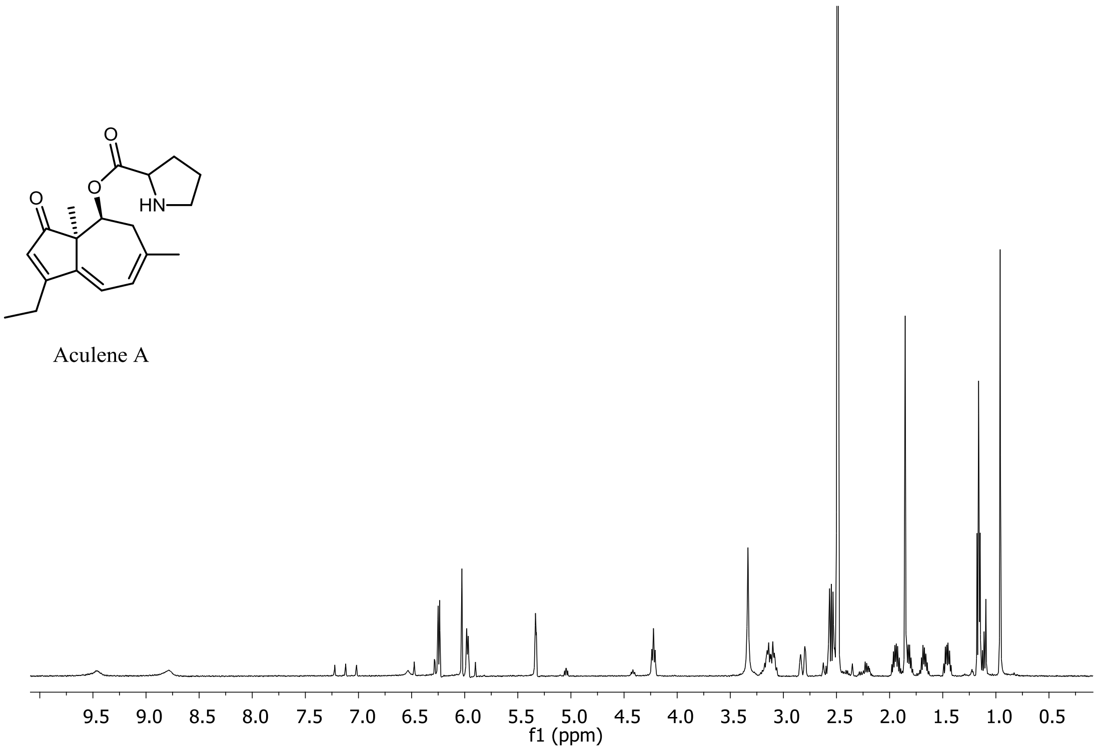

Figure S2. DQF-COSY spectrum of the new compound aculene A.

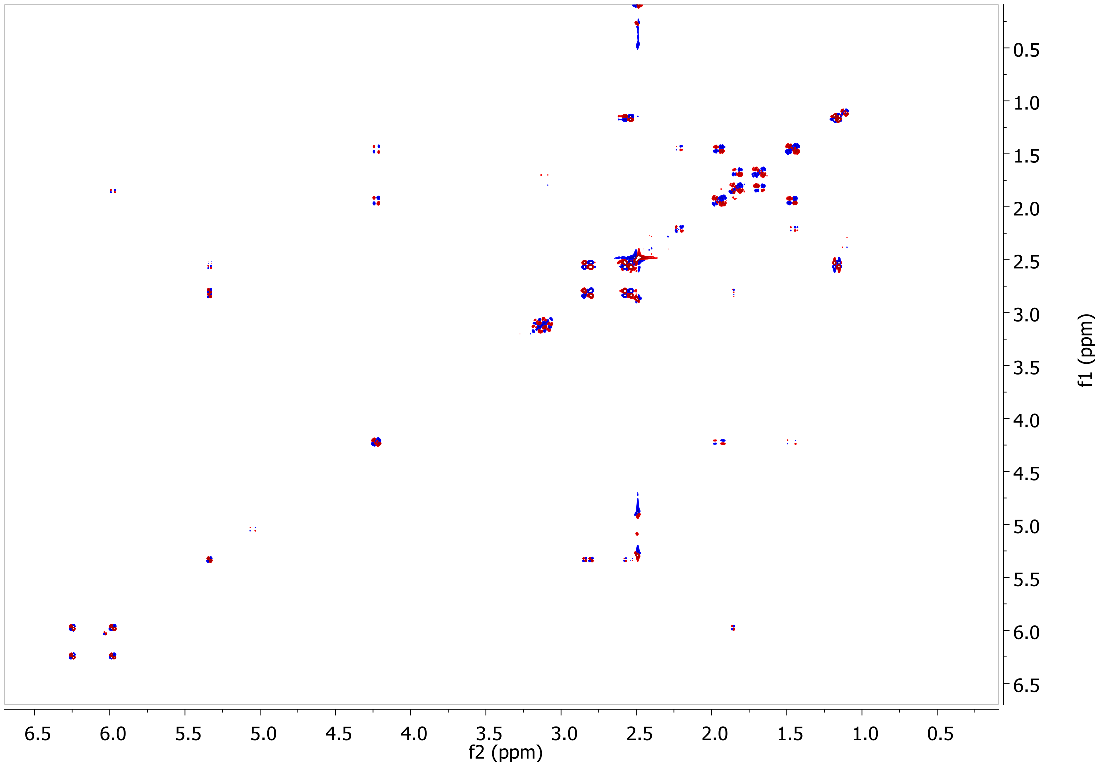

Figure S3. NOESY spectrum of the new compound aculene A.

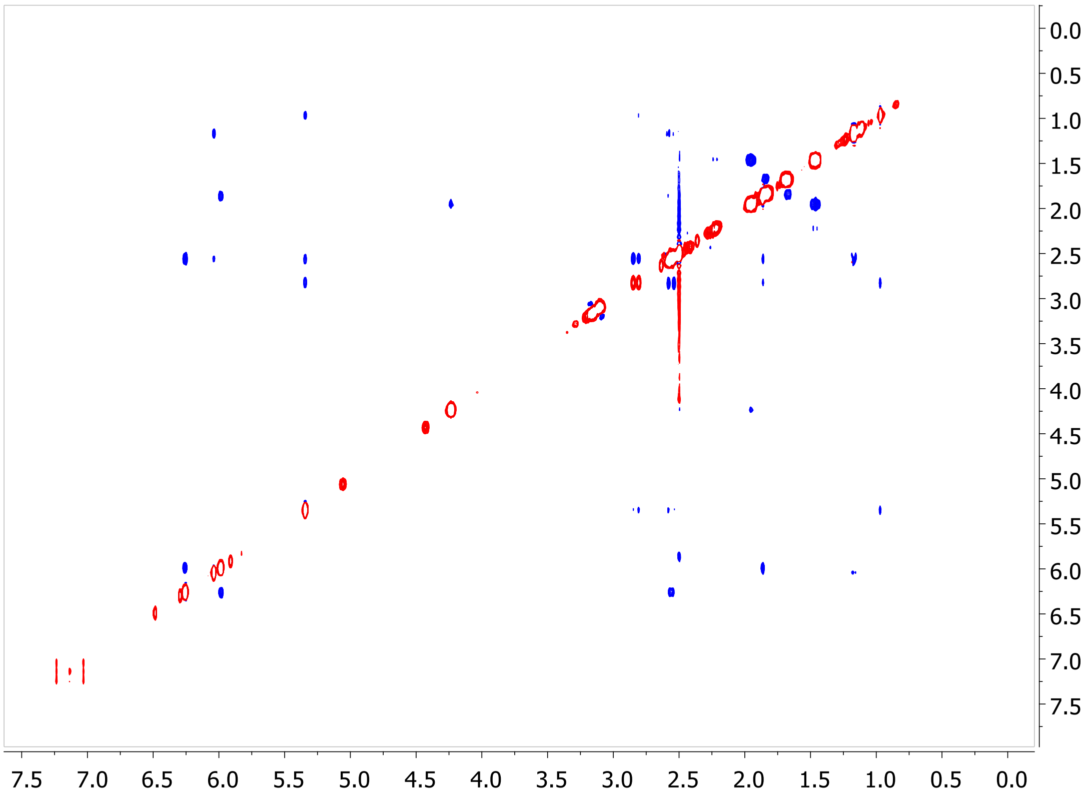

Figure S4. HSQC spectrum of the new compound aculene A.

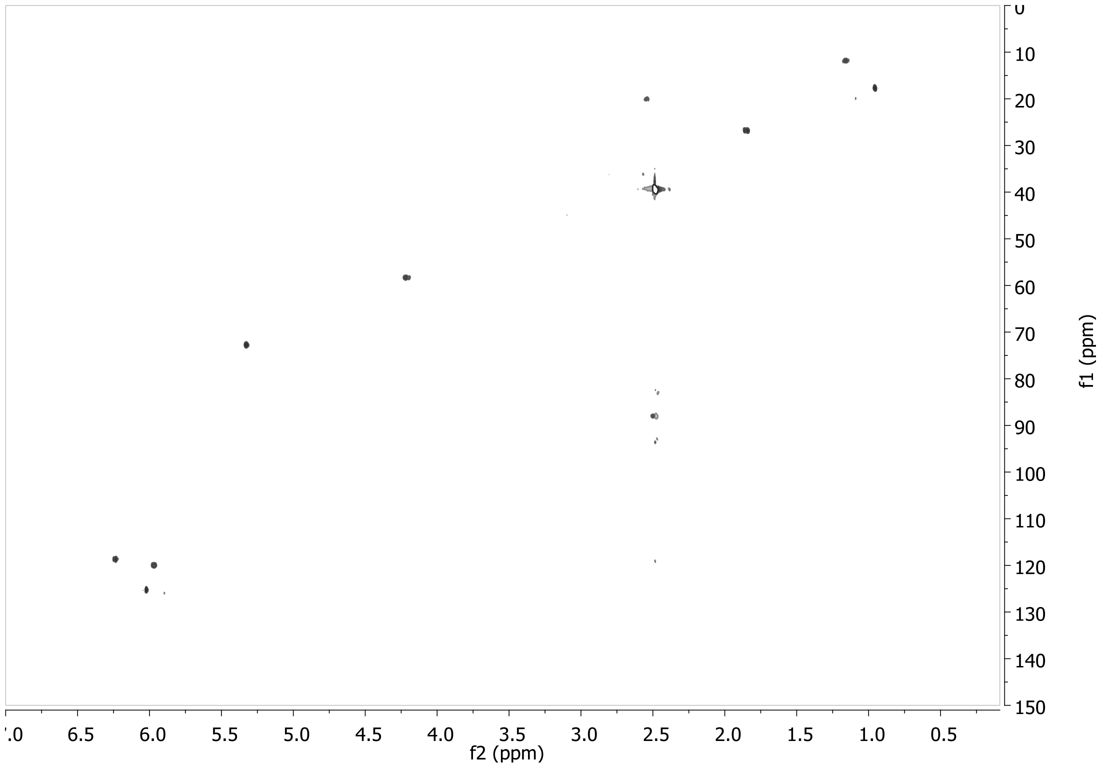

Figure S5. HMBC spectrum of the new compound aculene A.

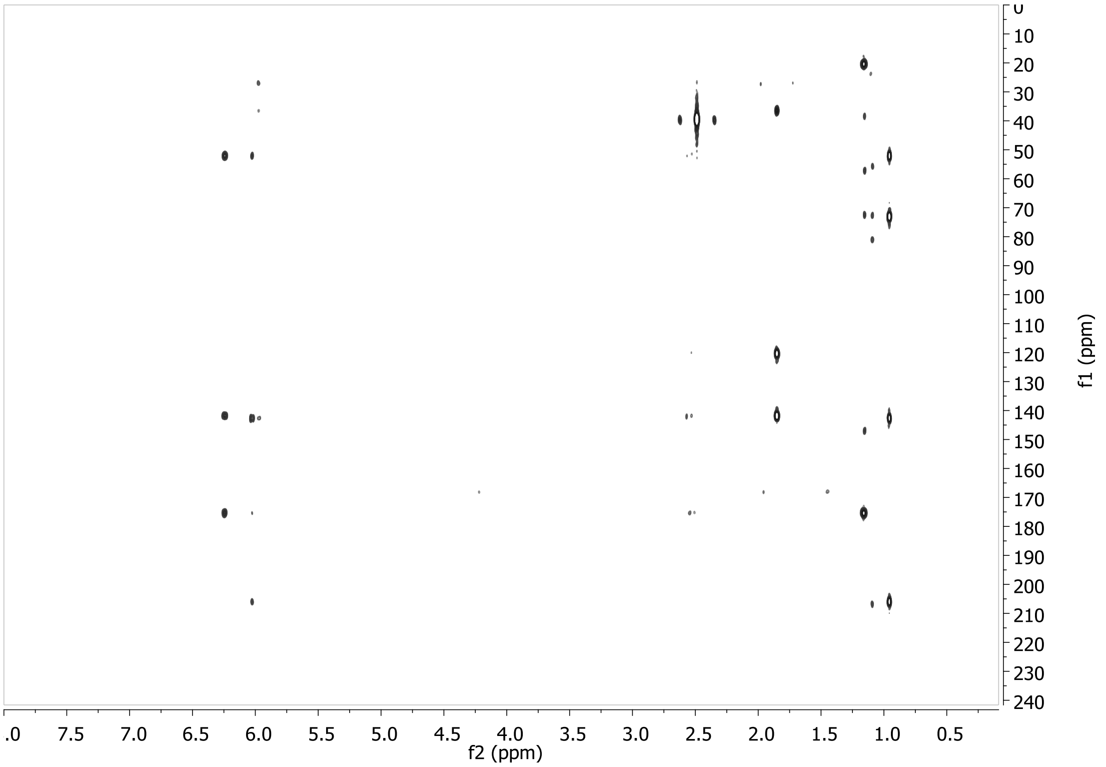

Figure S6. UV spectrum of the new compound aculene A.

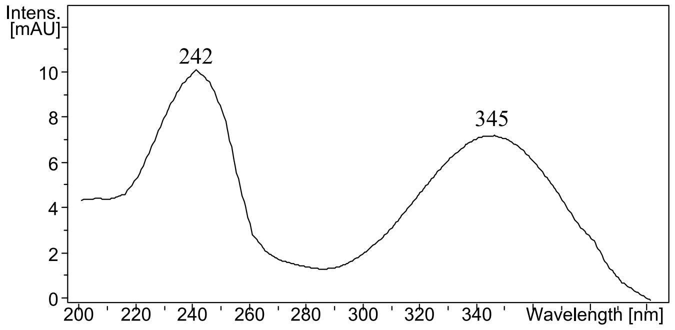

Figure S7. ^{1}H-NMR (500 MHz, DMSO-d_6 spectrum) of the new compound aculene B.

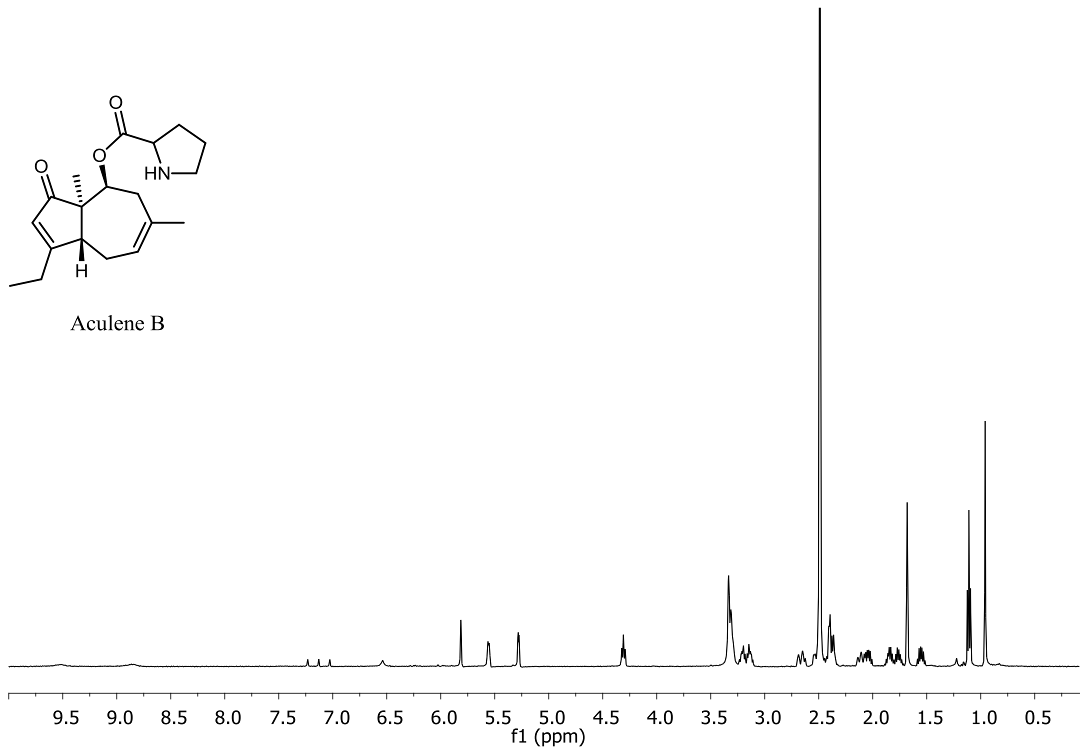

Figure S8. DQF-COSY spectrum of the new compound aculene B.

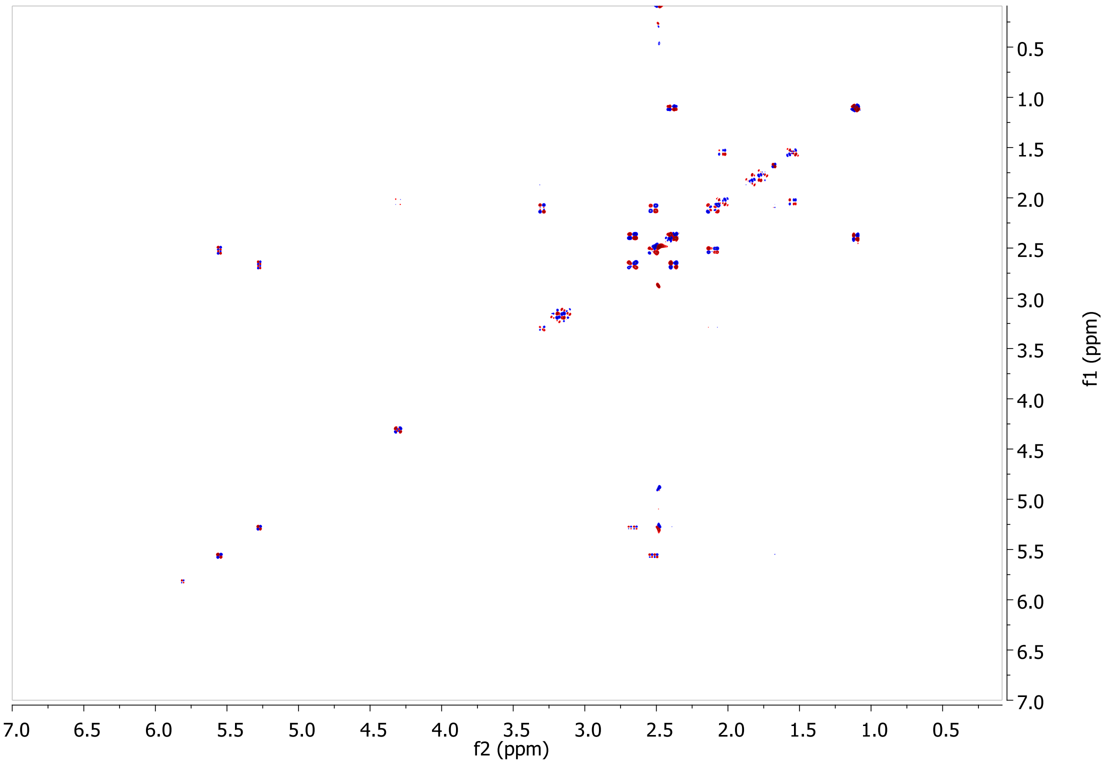

Figure S9. NOESY spectrum of the new compound aculene B.

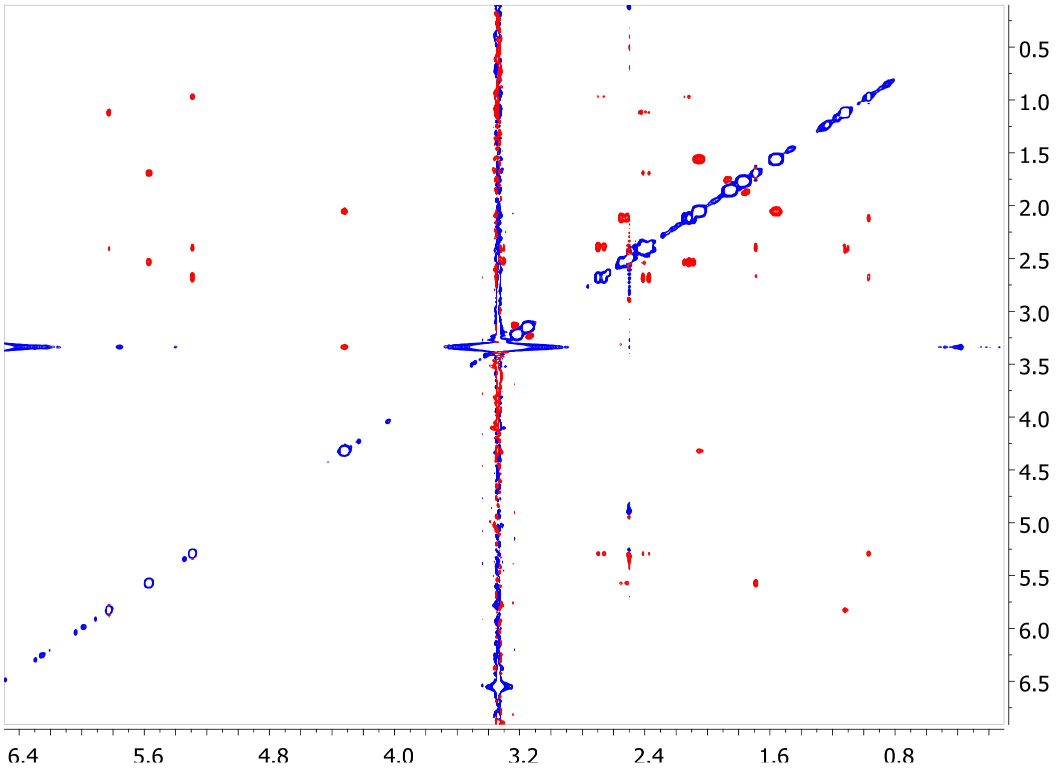

Figure S10. HSQC spectrum of the new compound aculene B

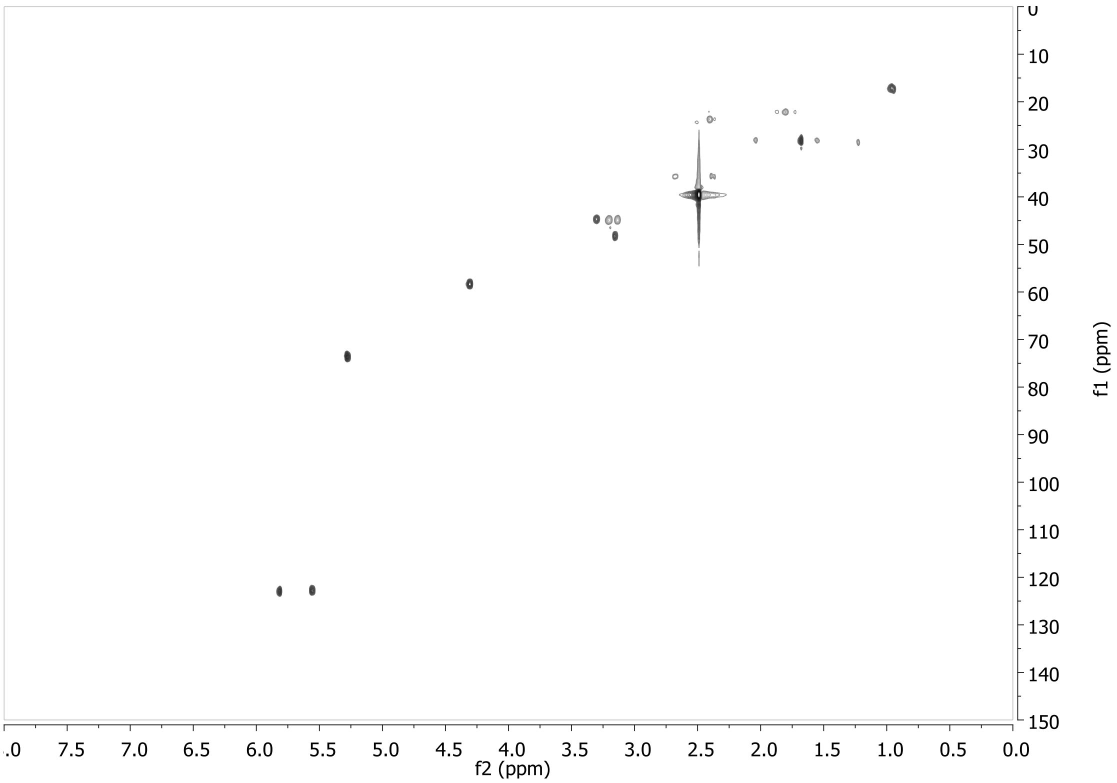

Figure S11. HMBC spectrum of the new compound aculene B.

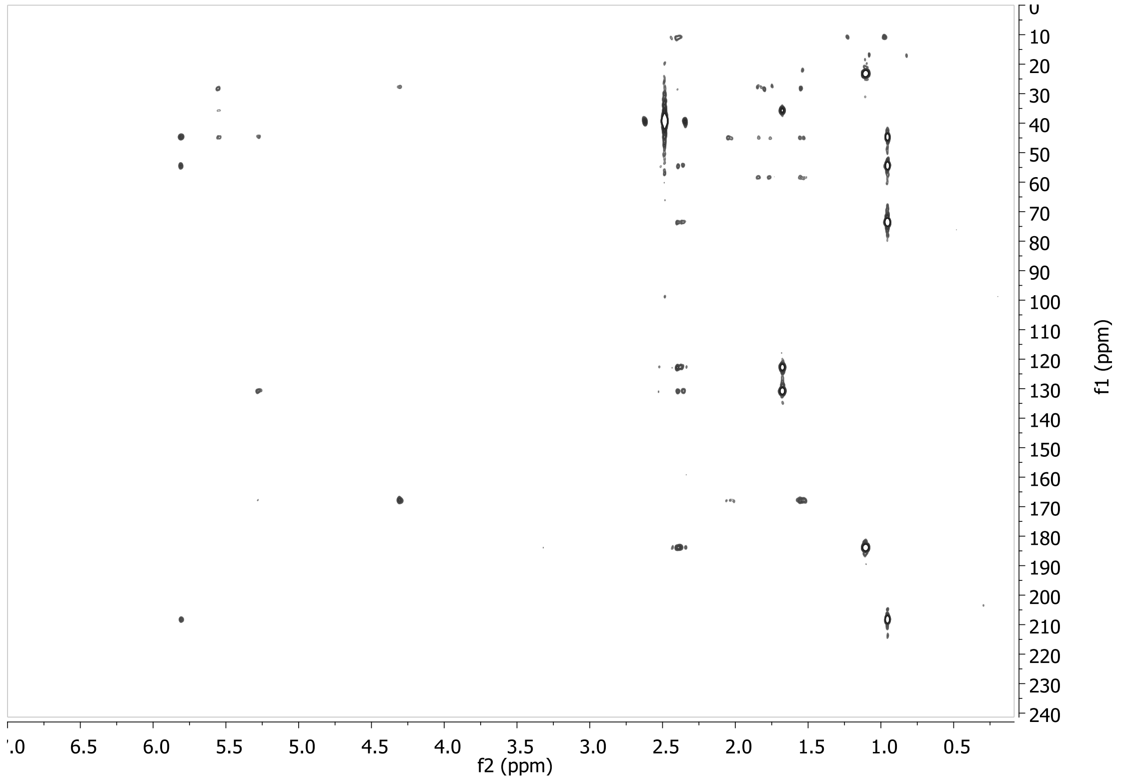

Figure S12. UV spectrum of the new compound aculene B.

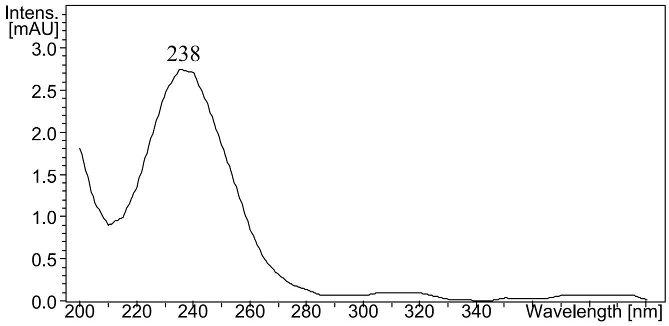

Figure S13. ^{1}H-NMR (800 MHz, DMSO-d_6 spectrum) of the new compound aculene C.

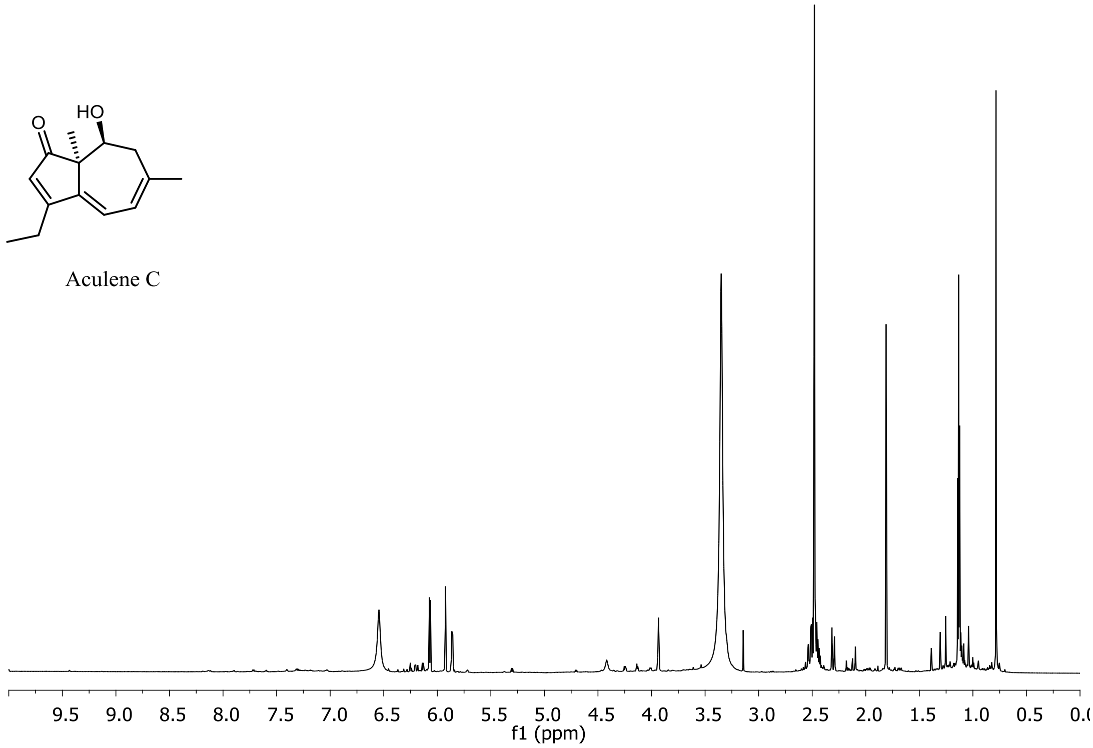

Figure S14. DQF-COSY spectrum of the new compound aculene C.

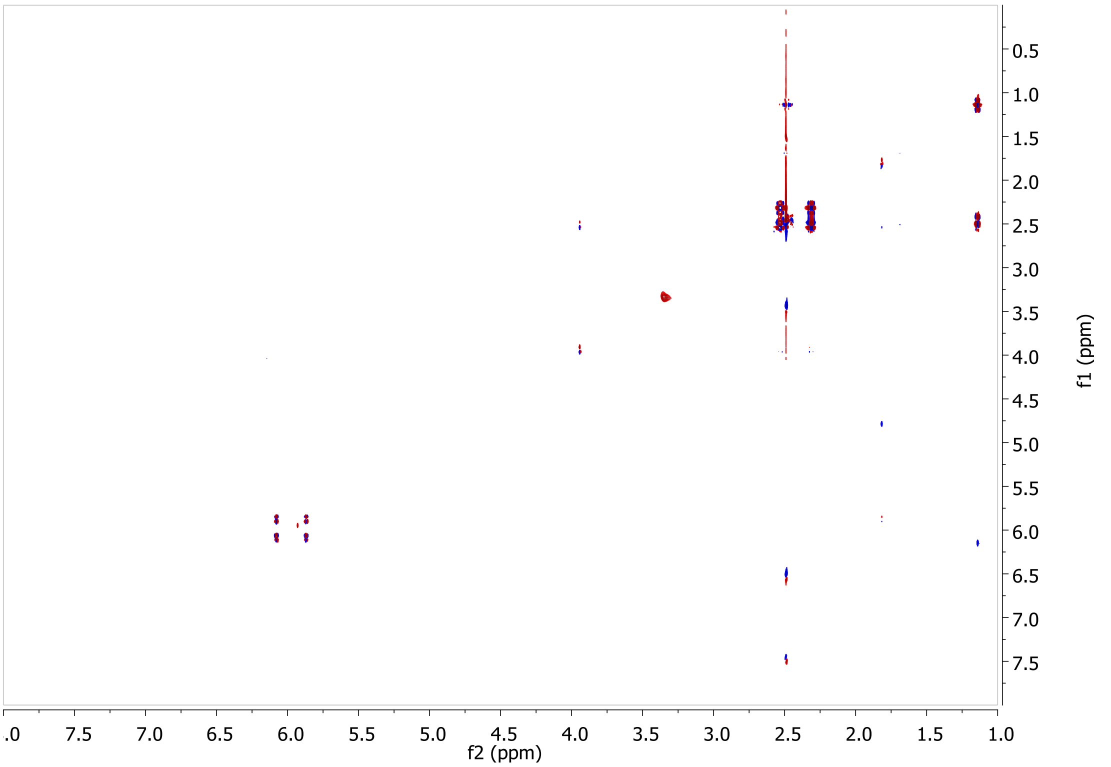

Figure S15. NOESY spectrum of the new compound aculene C.

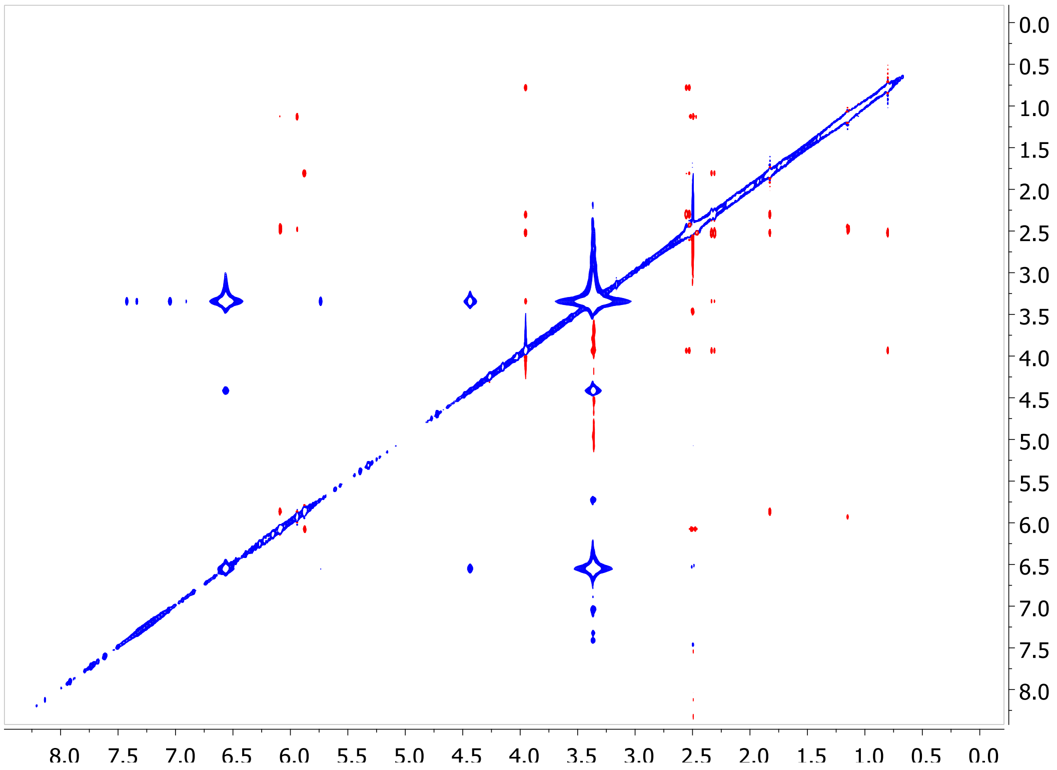

Figure S16. HSQC spectrum of the new compound aculene C.

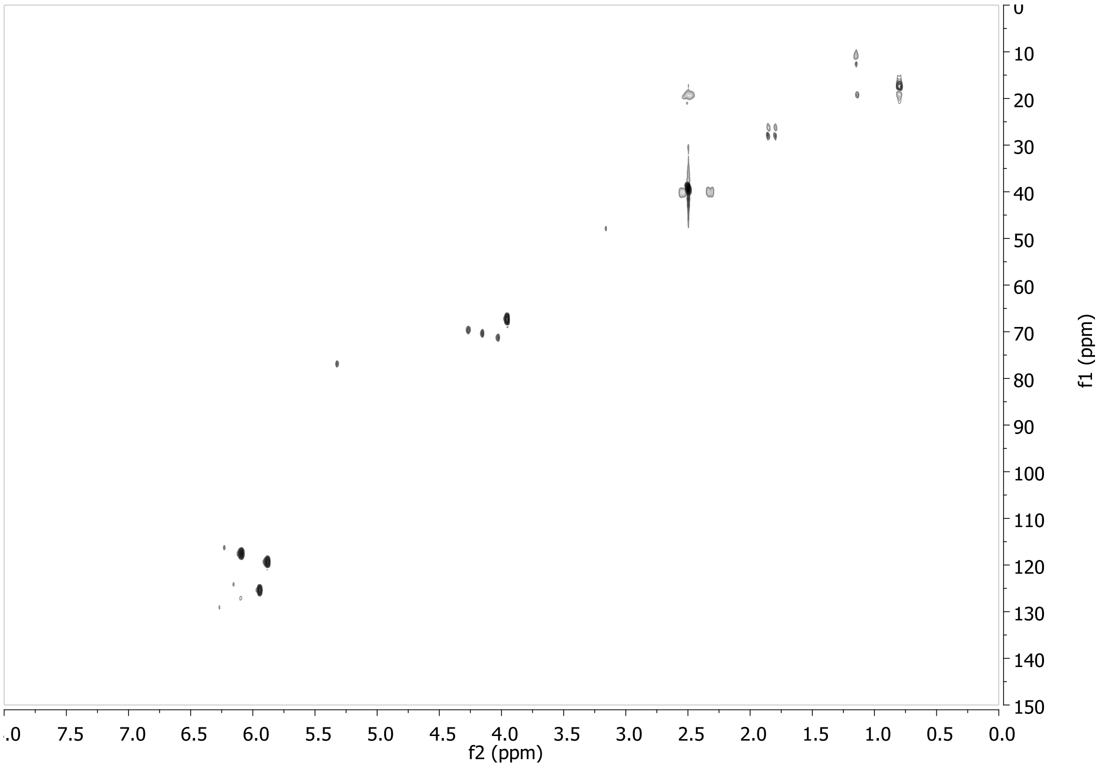

Figure S17. HMBC spectrum of the new compound aculene C.

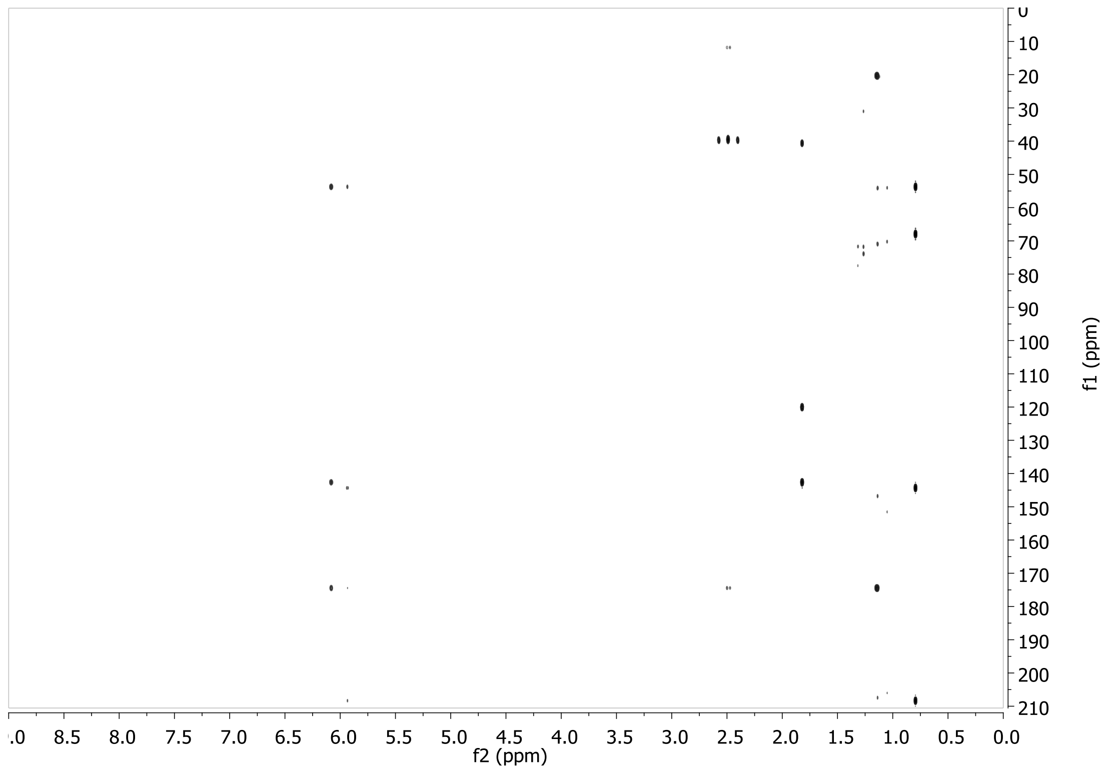

Figure S18.UV spectrum of the new compound aculene C.

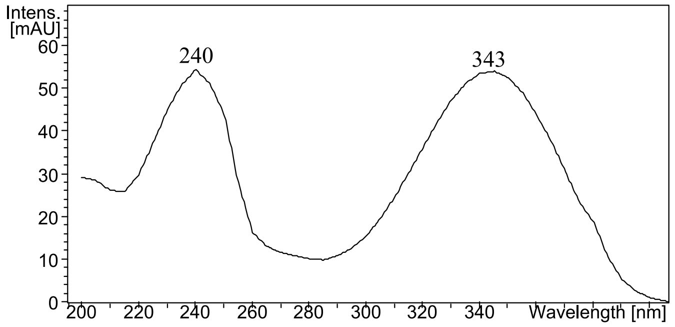

Figure S19. ^{1}H-NMR (500 MHz, DMSO-d_6 spectrum) of the new compound acu-dioxomorhpoline.

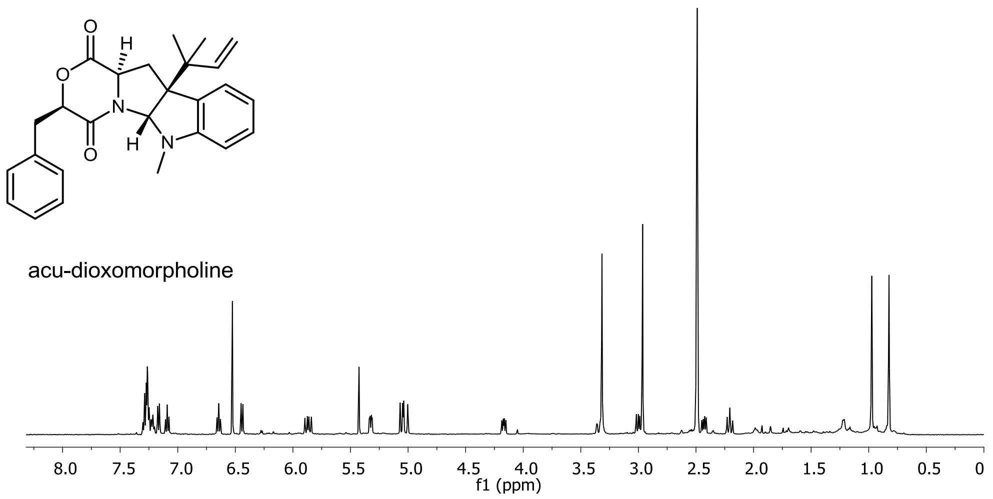

Figure S20. DQF-COSY spectrum of the new compound acu-dioxomorhpoline.

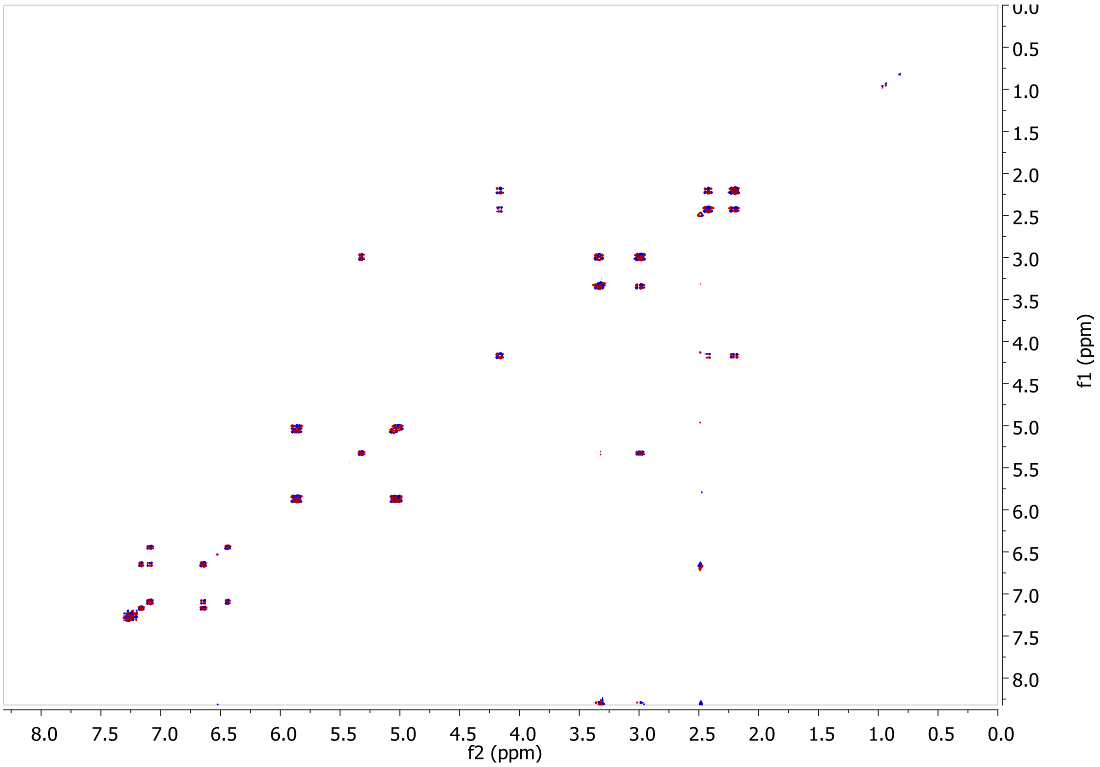

Figure S21. NOESY spectrum of the new compound acu-dioxomorhpoline.

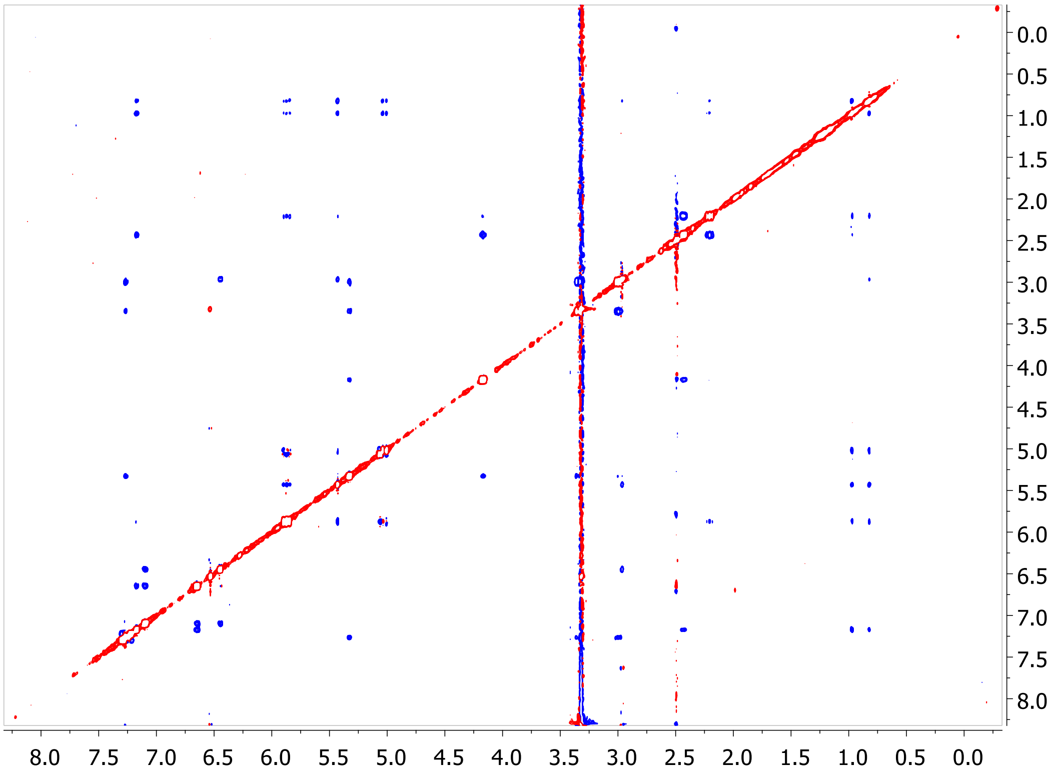

Figure S22. HSQC spectrum of the new compound acu-dioxomorhpoline.

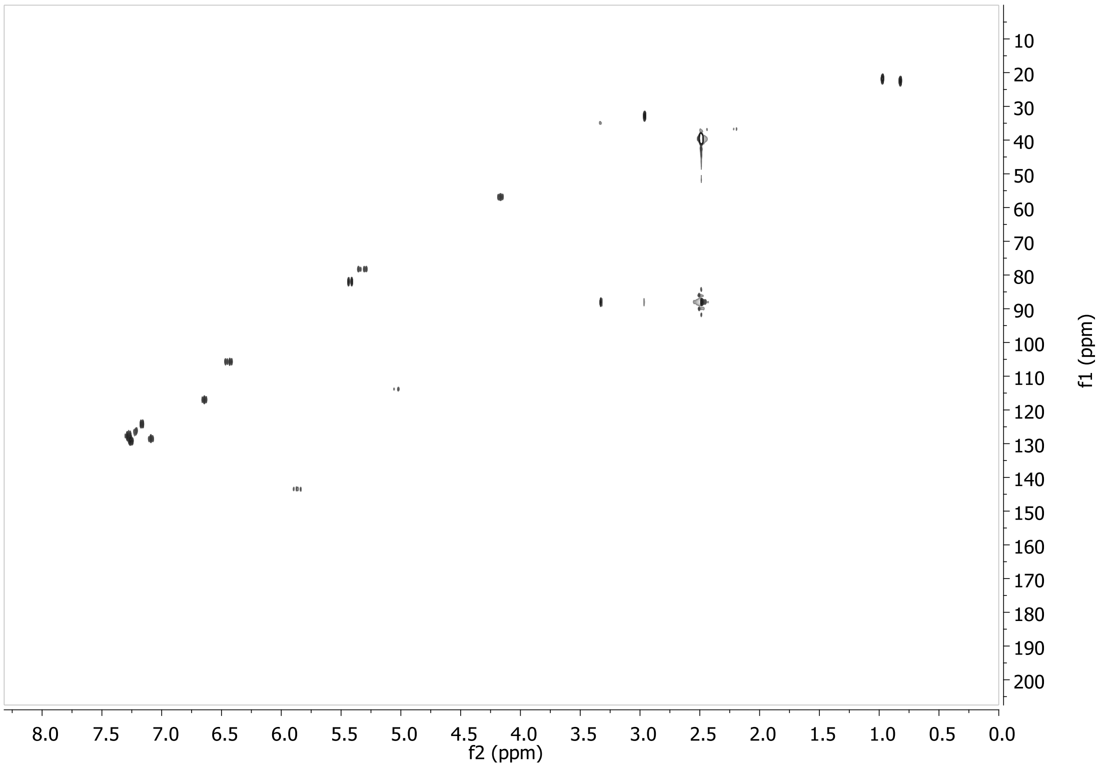

Figure S23. MBC spectrum of the new compound acu-dioxomorhpoline.

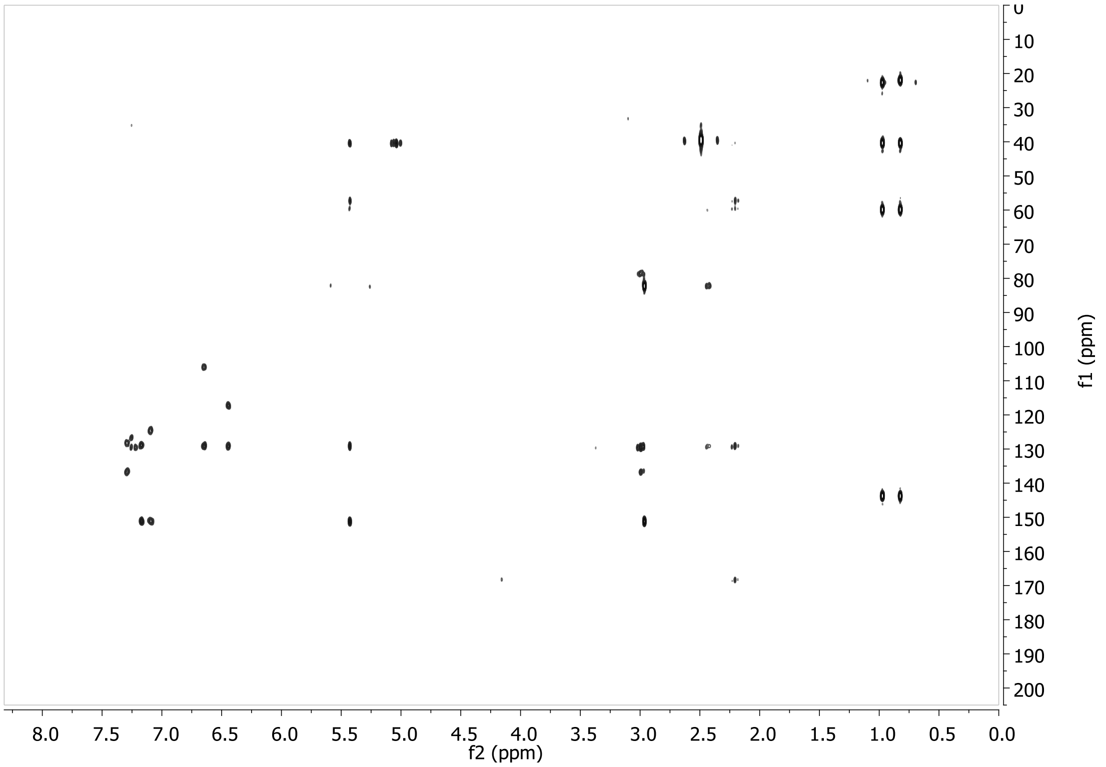

Figure S24. UV spectrum of the new compound acu-dioxomorhpoline.

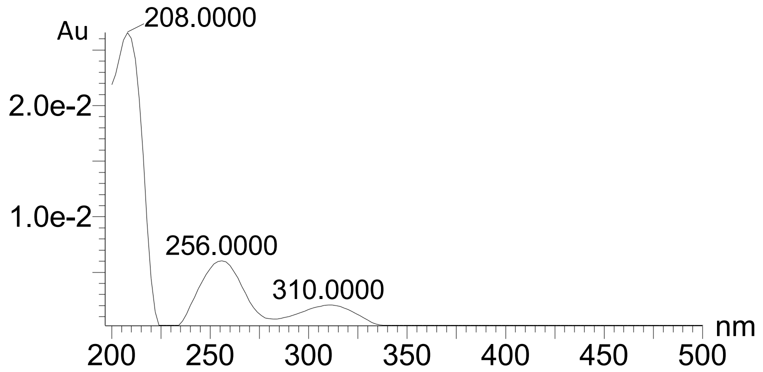

Figure S25. ^{1}H-NMR (500 MHz, DMSO-d_6 spectrum) of the new compound okaramine S.

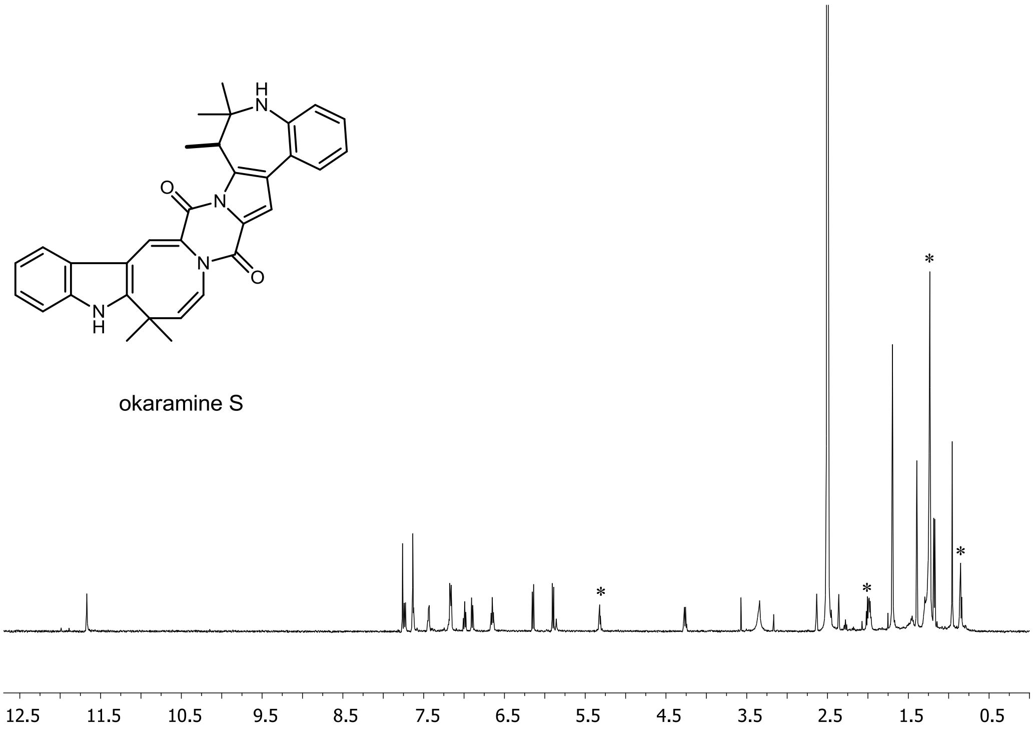

* Impurity (Isolated spinsystem, not part of the compound).

Figure S26. DQF-COSY spectrum of the new compound okaramine S.

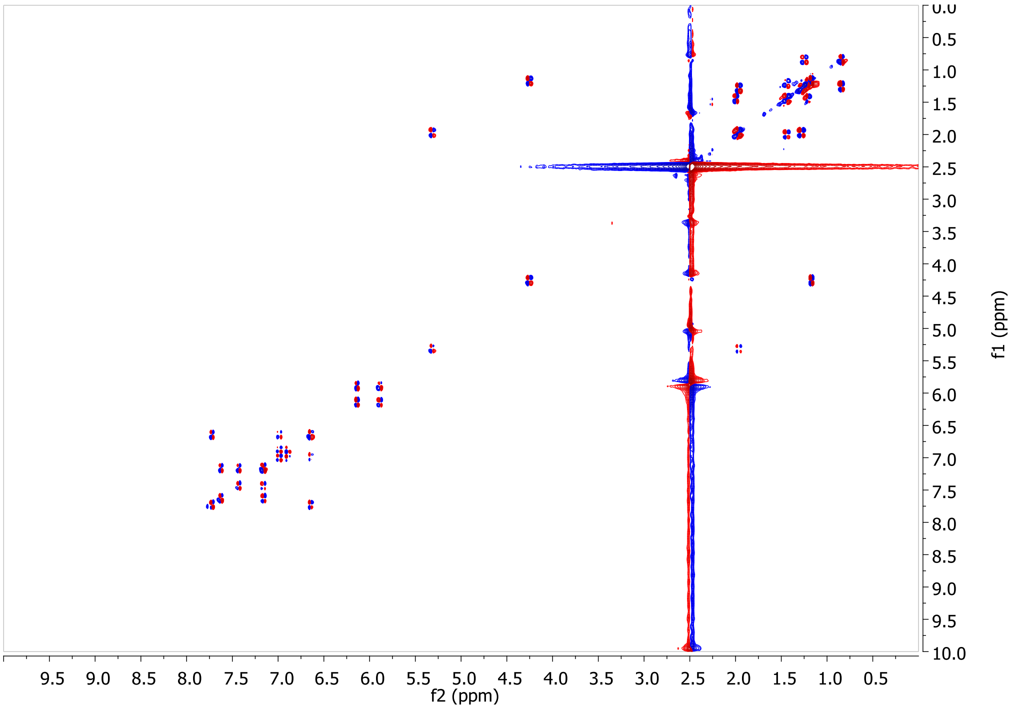

Figure S27. NOESY spectrum of the new compound okaramine S.

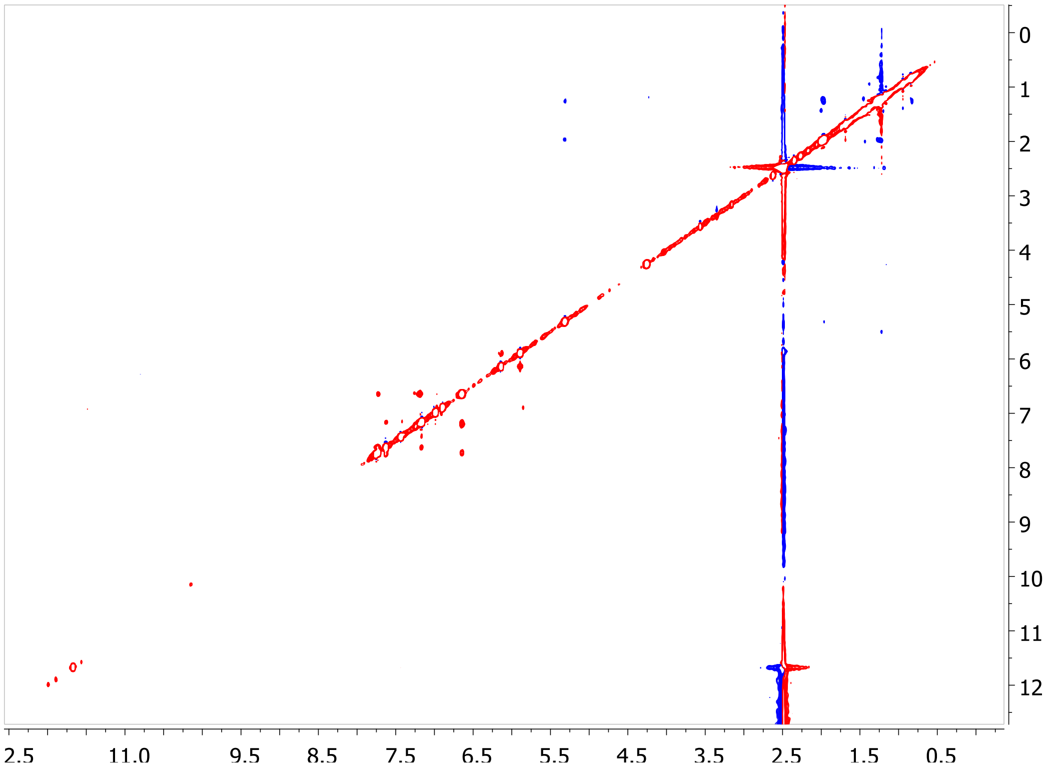

Figure S28. HSQC spectrum of the new compound okaramine S.

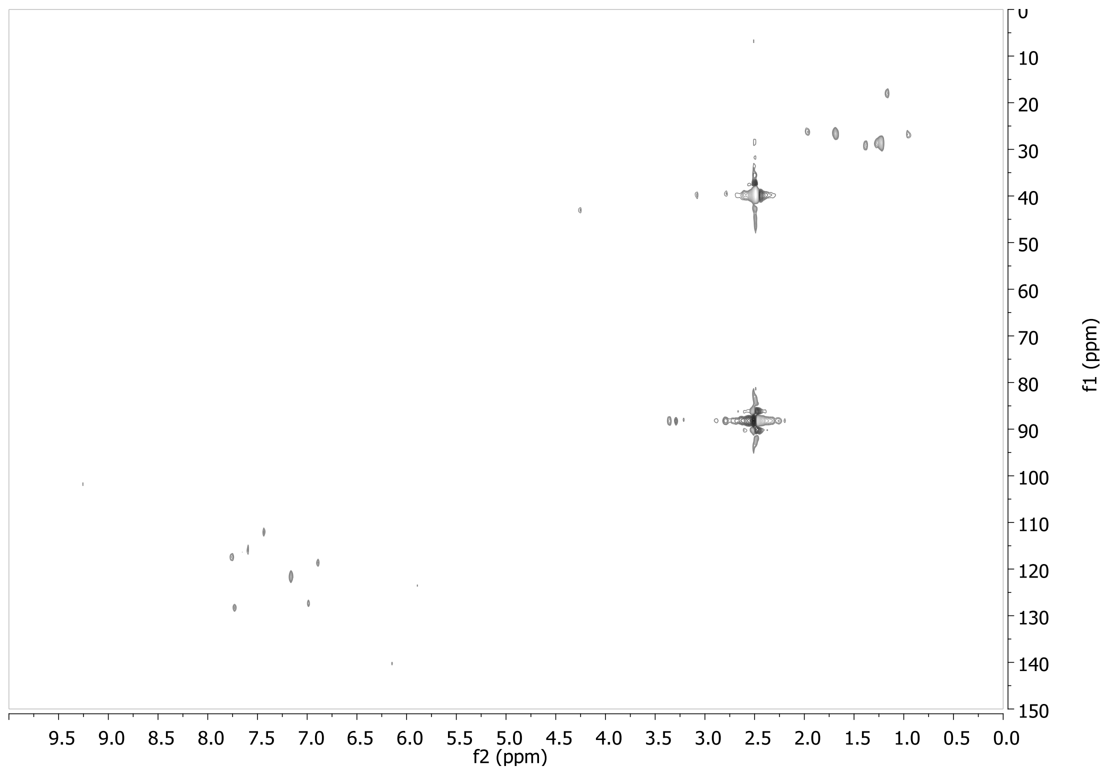

Figure S29. HMBC spectrum of the new compound okaramine S.

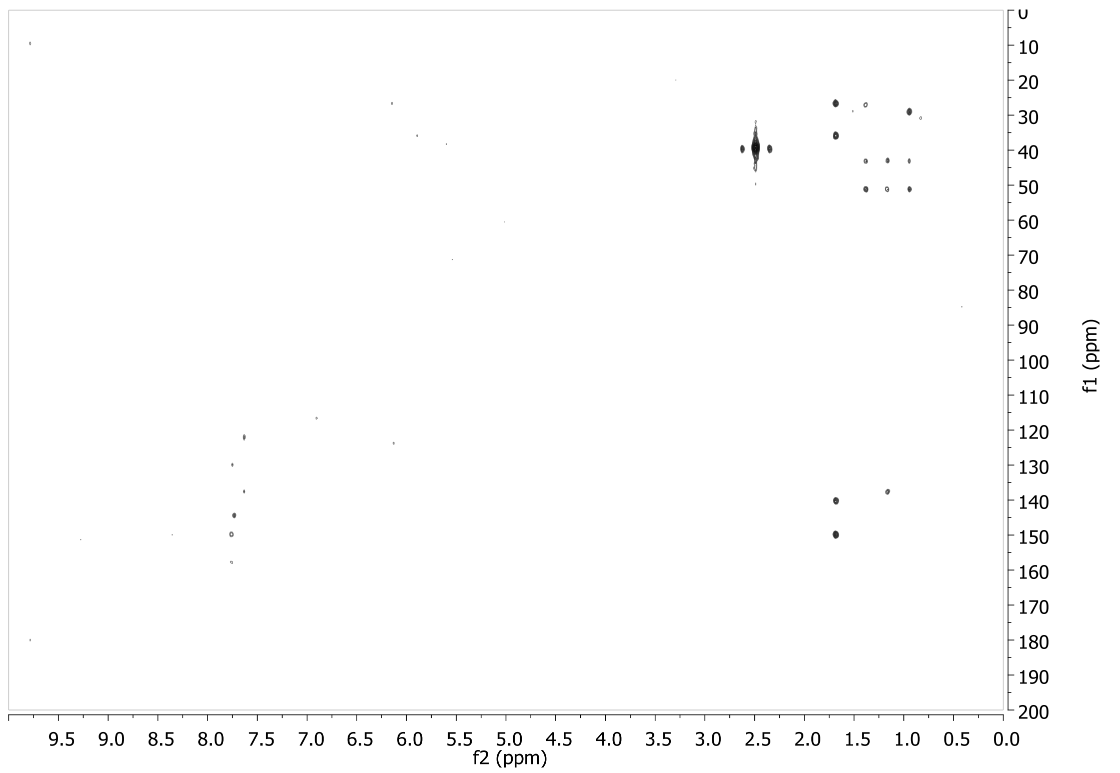

Figure S30. UV spectrum of the new compound okaramine S.

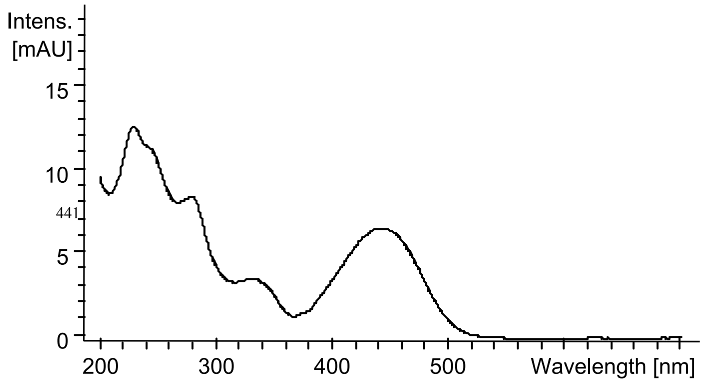

Figure S31.^{1}H-NMR (500 MHz, DMSO-d_6 spectrum) of epi-10,23-dihydro-24,25-dehydroaflavinine.

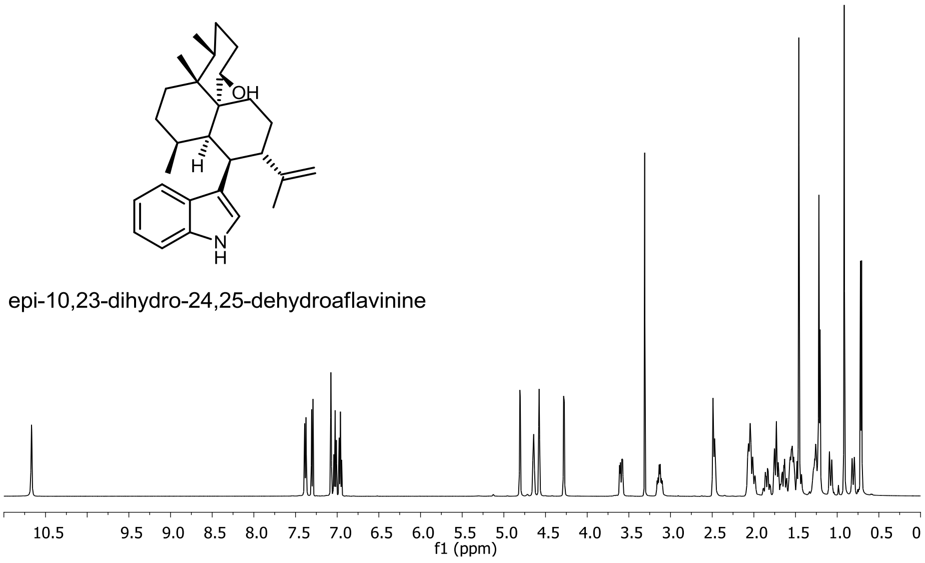

Figure S32. DQF-COSY spectrum of the new compound epi-10,23-dihydro-24,25-dehydroaflavinine.

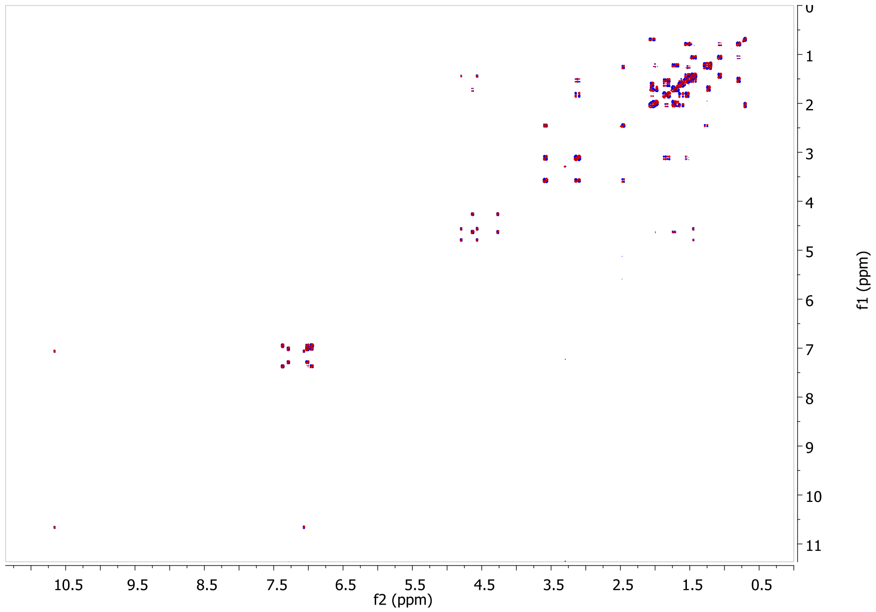

Figure S33. NOESY spectrum of the new compound epi-10,23-dihydro-24,25-dehydroaflavinine.

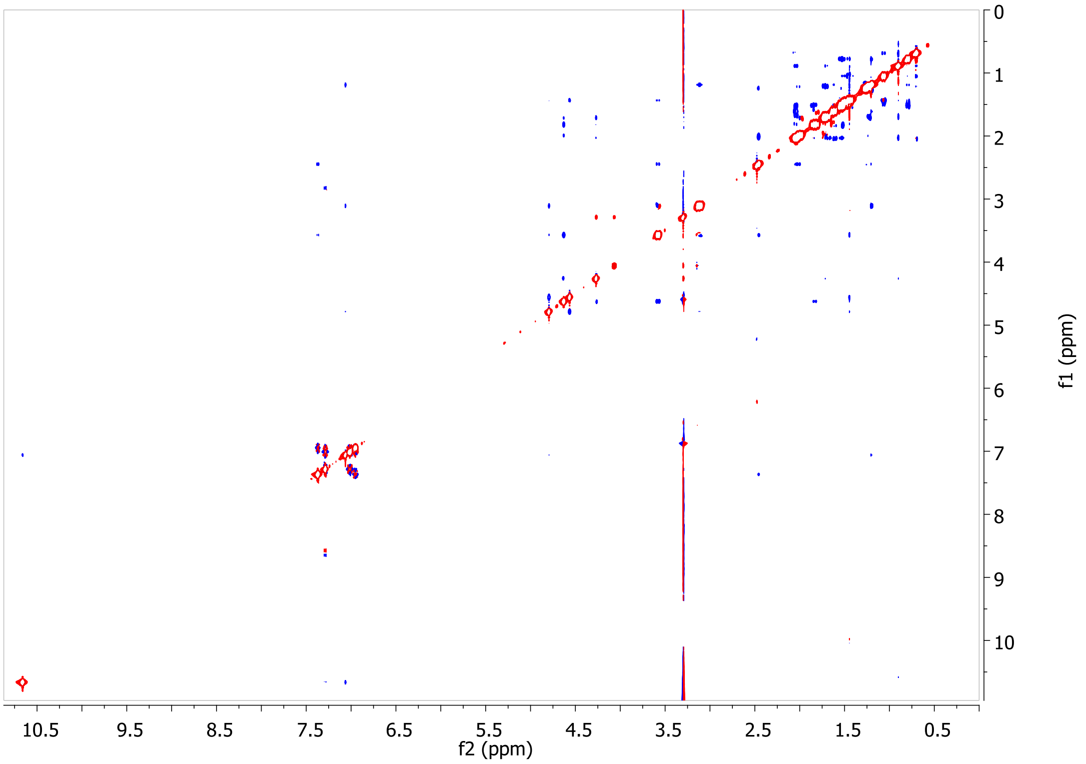

Figure S34. HSQC spectrum of the new compound epi-10,23-dihydro-24,25-dehydroaflavinine.

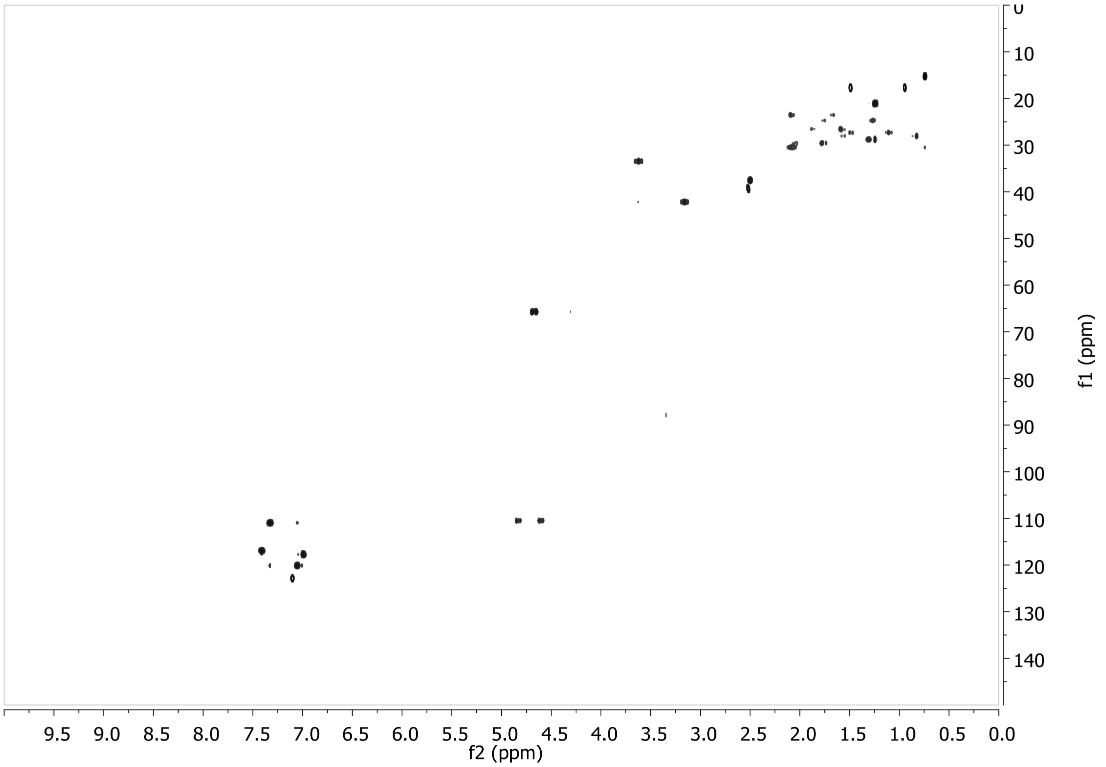

Figure S35. HMBC spectrum of the new compound epi-10,23-dihydro-24,25-dehydroaflavinine.

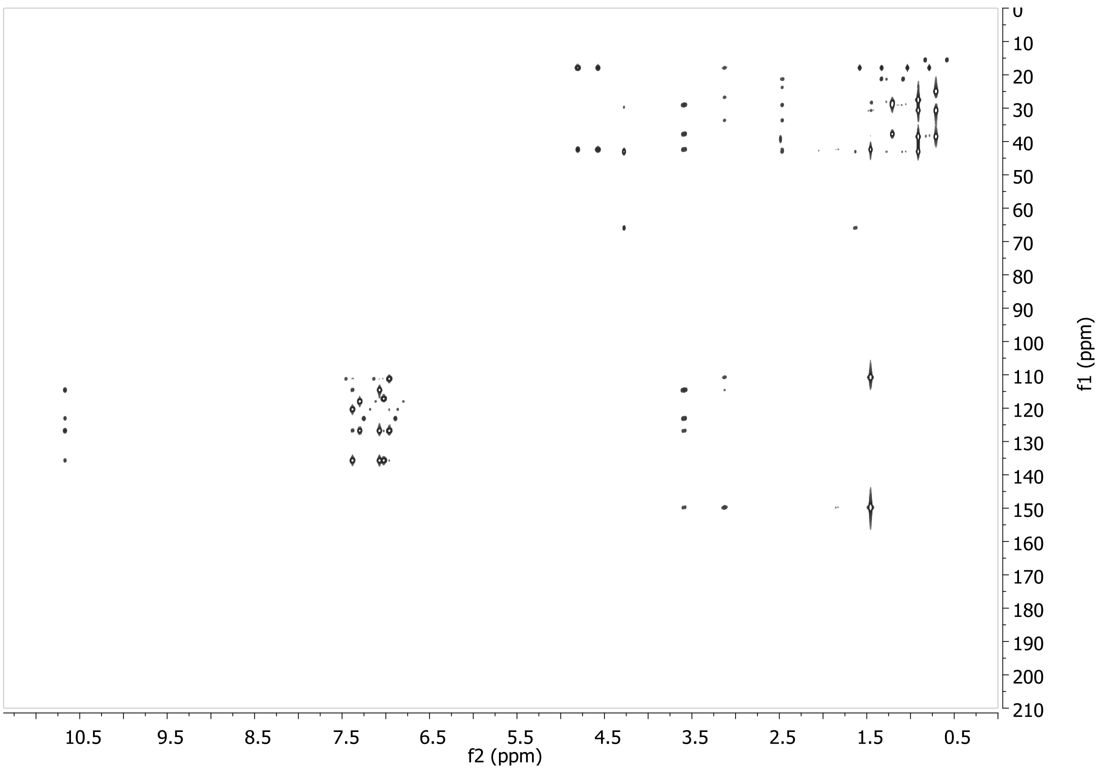

Figure S36. UV spectrum of the new compound epi-10,23-dihydro-24,25-dehydroaflavinine.

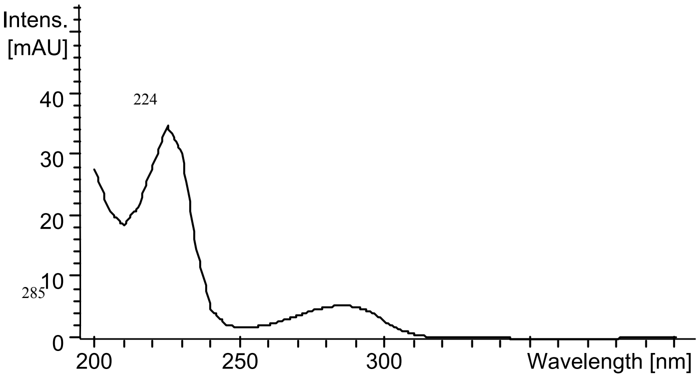

Figure S37. Selected NOEs for aculenes A and B.

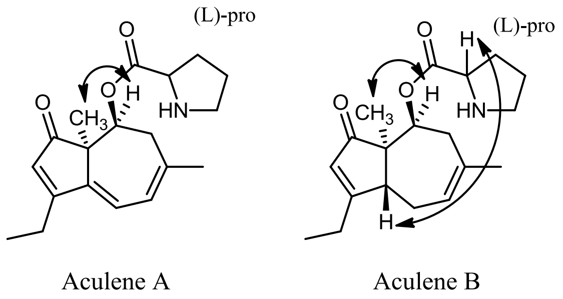

Stereochemistry of aculenes A–D: The relative stereochemistry of the stereocenters C-7 and C-16 was investigated by performing conformational searches on the two diastereomers of aculenes A-C.

The strain of the ring resulted in mainly two possible configurations per structure. Only the configuration which is seen in Figure S37 will result in the observed ^{3}J-coupling constants of H-7 as seen in Table S1.

Table S1 for aculene A. A similar trend was observed for aculenes B and C. The observed NOESY peaks (Table S2) fit the suggested structures the best. The final stereocenter (C-12) of aculene B is suggested from NOEs and from the rather large *J*-coupling from H-12 to H-11, which displays an NOE to H-17.

Table S1. Observed ^{3}J-couplings in Hz of H-7 of aculene A compared to values calculated by DFT.

Nucleus1	Nucleus2	Meas	(RS) or (SR) conf. 1	(RS) or (SR) conf. 2	(RR) or (SS) conf. 1	(RR) or (SS) conf. 2
7	8	4.4	4.5	11.0	9.3	11.3
7	8'	2.8	2.6	5.2	0.6	5.9

Table S2. NOESY correlations for aculenes A–C.

Position	Aculene A	Aculene B	Aculene C
1			
2		2'	
2'		2	
3	3'	3'	
3'	3	3,4'	
4	4',5	4',5	
4'	4	3',4	
5	4	4,12	
6			
7	8,8',17	8,8',17	8,8',17
8	7,8',17	7,8',17,18	7,8',17,18
8'	7,8	7,8	7,8,18
9			
10	11,18	11,11',18	11,18
11	10,19	10,11',17	10,19,20
11'		10,11	
12		5	
13			
14	19,20	17,19,20	17,19,20
15			
16			
17	7,8	7,8,11,14	7,8,14
18	10	8,10,19	8,8’,10
19	11,14,20	14,18,20	11,14,20
20	14,19	14,19	11,14,19
-OH			

Figure S38. Proposed structures of acucalbistrin A and B.

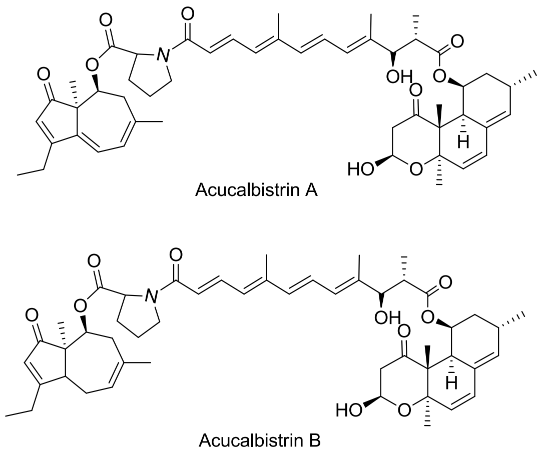

A. BPC and EICs of a fraction from attempted purification of acucalbistrin A. Fraction contains aculene A ($[M+H]^+$ = 316.1907), calbistrin A ($[M+Na]^+$ = 563.2615, ionizing poorly in positive ion mode) and acucalbistrin A ($[M+H]^+$ = 838.4524). Both acucalbistrin A and calbistrin A epimerize during purification. **B.** BPC and EICs of a fraction from attempted purification of acucalbistrin B. Fraction contains aculene B ($[M+H]^+$ = 318.2064), calbistrin A ($[M+Na]^+$ = 563.2615, ionizing poorly in positive ion mode) and acucalbistrin B ($[M+H]^+$ = 840.4681). Both acucalbistrin B and calbistrin A epimerize during purification.

Figure S39. Degradation of the acucalbistrins.

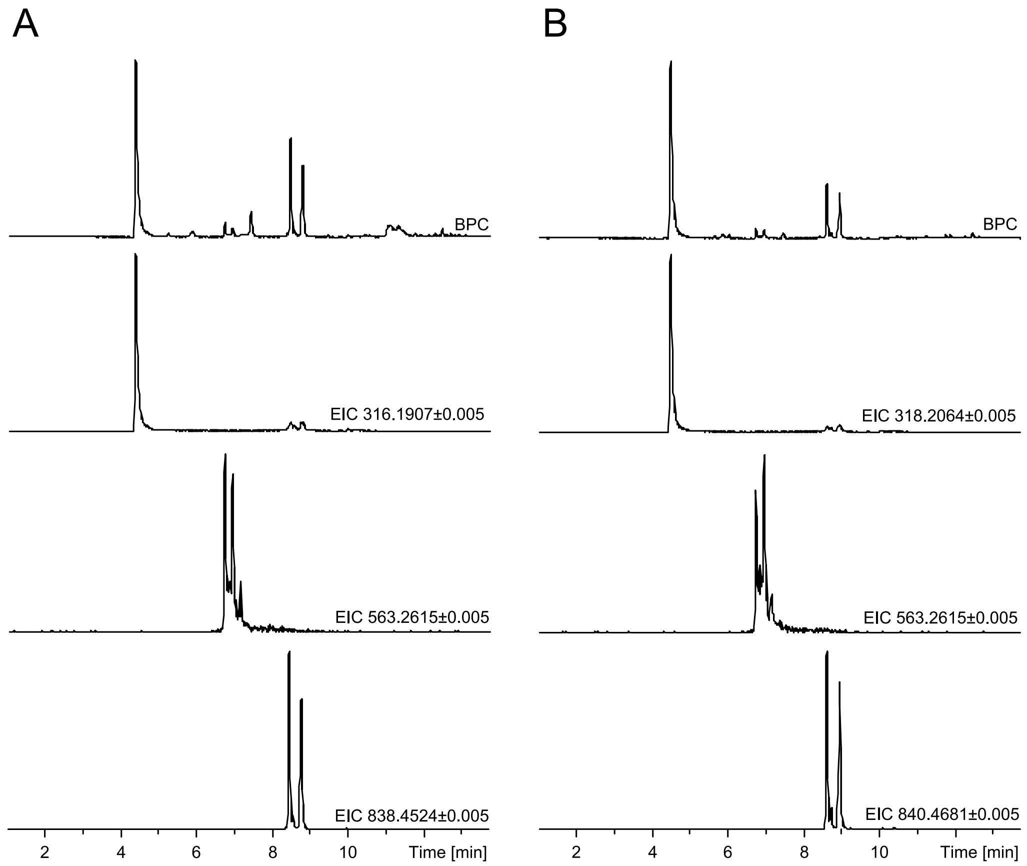

Figure S40. 3D-structure of 10,23-dihydro-24,25-dehydroaflavinine. Epi-10,23-dihydro-24,25-dehydroaflavinine is suggested to be the enantiomer

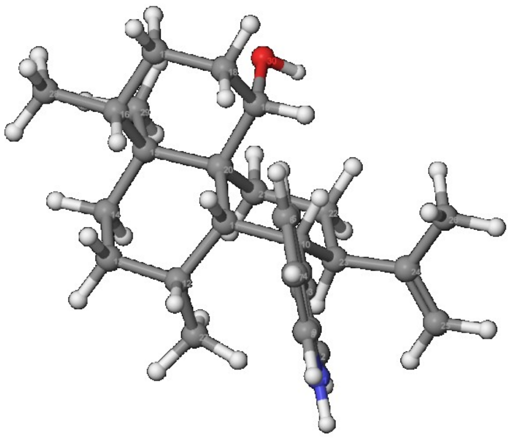

The relative stereochemistry of epi-10,23-dihydro-24,25-dehydroaflavinine was established by performing conformational searches on different diastereomers and comparing observed distances to the back-calculated distances from ISPA, as well as comparing ^{3}J-couplings. The structure which fitted the data the best was further optimized by HF and DFT to give the reported structure.

Table S3. ISPA derived distances in Å of epi-10,23-dihydro-24,25-dehydroaflavinine, a: reference, b: error due to freely rotating bond.

Nucleus1	Nucleus2	Meas	Calc	Diff	Lower bound	Violation	Upper bound	Violation
1	2	2.50	2.64	0.14	2.38		2.90	
1	5	2.83	3.03	0.20	2.72		3.33	
5	18	2.40	2.72	0.32	2.45	0.05	2.99	
5	11	2.37	2.41	0.04	2.17		2.65	
5	10	2.67	2.54	0.13	2.29		2.79	
2	27	2.54	2.48	0.06	2.23		2.73	
2	23	2.46	2.58	0.12	2.32		2.83	
2	25	2.96	3.01	0.05	2.71		3.32	
25	23	2.31	2.39	0.07	2.15		2.62	
25	10	4.58	2.78	1.80[b]	2.51		3.06	1.52
25	25'	1.85	1.85	0.00[a]	1.66		2.03	
19	26	2.95	2.98	0.04	2.68		3.28	
19	18'	2.43	2.51	0.08	2.25		2.76	
19	22	2.14	2.02	0.12	1.82		2.22	
19	18	2.41	2.32	0.08	2.09		2.56	
19	11	2.83	3.37	0.53	3.03	0.20	3.70	
19	10	1.88	1.95	0.07	1.75		2.14	
30	29	2.83	2.88	0.05	2.59		3.17	
30	18'	3.32	2.48	0.84[b]	2.23		2.73	0.59
30	22	2.25	2.68	0.44[b]	2.41	0.16	2.94	
30	21	2.06	2.65	0.59[b]	2.39	0.33	2.92	

Table S3. *Cont.*

Nucleus1	Nucleus2	Meas	Calc	Diff	Lower bound	Violation	Upper bound	Violation
10	26	2.33	2.28	0.05	2.05		2.50	
10	11	2.32	2.24	0.08	2.02		2.47	
23	27	2.05	2.06	0.02	1.86		2.27	
11	12	2.24	2.12	0.12	1.90		2.33	
11	13	2.61	2.70	0.09	2.43		2.97	
11	16	2.21	1.86	0.35	1.68		2.05	0.16
16	28	2.37	2.23	0.14	2.01		2.46	
21	29	2.18	2.05	0.12	1.85		2.26	
16	17'	2.44	2.10	0.34	1.89		2.31	0.13
16	13	2.02	1.93	0.09	1.73		2.12	
22	22'	1.74	1.75	0.01	1.58		1.93	
17	29	2.33	2.31	0.02	2.08		2.54	
17	17'	1.75	1.93	0.18	1.74		2.13	
21'	27	2.07	2.12	0.04	1.91		2.33	
13	13'	1.75	1.75	0.00	1.57		1.92	
17'	28	2.46	2.35	0.11	2.12		2.59	
14'	28	2.16	2.11	0.04	1.90		2.33	
25’	26	2.44	2.38	0.06	2.14		2.62	

Table S4. HLA and DFT ^{3}J-coupling constants in Hz of epi-10,23-dihydro-24,25-dehydroaflavinine.

Nucleus1	Nucleus2	Meas	Calc (HLA)	Diff	Calc (DFT)	Diff
10	23	13.3	11.1	2.2	12.4	0.9
10	11	5.0	5.4	0.4	5.1	0.1
21'	22	13.5	11.8	1.7	11.9	1.6
22	23	13.5	10.6	2.8	13.7	0.2
16	17	11.3	11.4	0.1	13.7	0.2
17	18	11.3	12.1	0.8	14.1	2.8
21'	22'	3.4	4.9	1.5	4.4	1
21	22	4.3	4.6	0.3	4.4	0.1
22'	23	5.6	5.4	0.2	5.3	0.3
